# Searching for Monomeric Nickel Tetrafluoride: Unravelling Infrared Matrix Isolation Spectra of Higher Nickel Fluorides

**DOI:** 10.1002/anie.202015501

**Published:** 2021-01-28

**Authors:** Lin Li, Ahmed K. Sakr, Tobias Schlöder, Siri Klein, Helmut Beckers, Marios‐Petros Kitsaras, Howard V. Snelling, Nigel A. Young, Dirk Andrae, Sebastian Riedel

**Affiliations:** ^1^ Freie Universität Berlin Institut für Chemie und Biochemie—Anorganische Chemie Fabeckstrasse 34/36 14195 Berlin Germany; ^2^ Department of Chemistry and Biochemistry University of Hull Kingston upon Hull HU6 7RX UK; ^3^ Karlsruher Institut für Technologie Institut für Nanotechnologie Hermann-von-Helmholtz-Platz 1 76344 Eggenstein-Leopoldshafen Germany; ^4^ Freie Universität Berlin Institut für Chemie und Biochemie—Theoretische Chemie Arnimallee 22 14195 Berlin Germany; ^5^ Department of Physics and Mathematics University of Hull Kingston upon Hull HU6 7RX UK

**Keywords:** IR spectroscopy, matrix isolation, nickel fluorides, tetrafluoride, trifluoride

## Abstract

Binary transition metal fluorides are textbook examples combining complex electronic features with most fundamental molecular structures. High‐valent nickel fluorides are among the strongest known fluorinating and oxidizing agents, but there is a lack of experimental structural and spectroscopic investigations on molecular NiF_3_ or NiF_4_. Apart from their demanding synthesis, also their quantum‐chemical description is difficult due to their open shell nature and low‐lying excited electronic states. Distorted tetrahedral NiF_4_ (*D*_2d_) and trigonal planar NiF_3_ (*D*_3h_) molecules were produced by thermal evaporation and laser ablation of nickel atoms in a fluorine/noble gas mixture and spectroscopically identified by a joint matrix‐isolation and quantum‐chemical study. Their vibrational band positions provide detailed insights into their molecular structures.

## Graphical abstract

Molecular NiF_3_ and NiF_4_ are the missing tri‐ and tetrafluorides of the otherwise known binary first‐row transition‐metal fluorides that are predicted to be stable. These elusive high‐valent molecular nickel fluorides were identified in rare‐gas matrices aided by high‐level quantum chemical electronic structure calculations. They are among the most powerful known fluorination and oxidation agents.

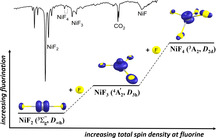

Binary fluorides of the late 1^st^ row transition metals (TM) are among the most efficient oxidative fluorinating agents, especially for the production of perfluorinated organic compounds.^[1]^ CoF_3_ is of industrial importance in the Flutec or Fowler processes,^[2]^ and higher nickel fluorides are considered to be the active fluorinating agents in the electrochemical fluorination of organic substrates (Simons process).^[3]^ Despite numerous attempts to elucidate this latter process the active species are still unknown.[^3^, ^4^]

Clearly, these particular properties are related to the peculiar electronic structure of these late TM fluorides, such as high ionization energies, the occupation of M−F antibonding molecular orbitals, and the lack of the so‐called “primogenic repulsion”, caused by the absence of radial nodes in the 3*d* valence orbitals.^[5]^ Nickel is one of the most electronegative metallic elements.^[6]^ This implies a considerable covalent character of the Ni−F bonds, which increases with the oxidation state of nickel.[^7^, ^8^] Particularly for high‐valent 3*d* TM complexes the similar radial extent of the 3*d* valence and the 3*p* core shell leads to repulsion between core 3*p* and ligand valence electrons, which ultimately results in weakened metal‐ligand bonds,^[5]^ a weak ligand‐field, and the presence of several low‐lying excited electronic (spin) states.^[9]^ Taken together, these circumstances represent a challenge for quantum‐chemical predictions and a major impediment for the spectral analysis of the still elusive high‐valent molecular nickel fluorides.

In addition to the trifluorides, the tetrafluorides of the 1^st^ row TM are particularly interesting. Solid MnF_4_ releases elemental fluorine at temperatures >170 °C.^[10]^ It can be used for storage and purification of elemental fluorine.^[2]^ In contrast to solid MnF_4_ and NiF_4_, solid FeF_4_ or solid CoF_4_ are unknown, while the monomeric FeF_4_ and CoF_4_ species have been investigated by high‐temperature vapor‐phase mass spectrometry and cryogenic matrix‐isolation spectroscopy.[^11^, ^12^, ^13^]

Solid NiF_4_ is one of the strongest known fluorinating oxidizing agents,^[2]^ but it is poorly characterized spectroscopically and even its solid‐state structure is unknown. We have not found any report on molecular or gaseous NiF_4_.^[4]^ Fluorination of solid NiF_2_ provides thermally unstable higher nickel fluorides such as Ni_2_F_5_ (Ni^II^_3_Ni^IV^F_10_)^[14]^ or R‐NiF_3_ (Ni^II^Ni^VI^F_6_),^[15]^ and neutral NiF_4_ is claimed to be formed at about −60 °C by treating [NiF_6_]^2−^ salts in anhydrous HF with strong Lewis acids such as AsF_5_, SbF_5_, or BF_3_.^[16]^ NiF_4_ releases F_2_ above −55 °C and forms NiF_3_, which again releases fluorine above 20 °C to form NiF_2_.^[17]^ As far as we know molecular NiF_3_ has not yet been studied spectroscopically, but it has attracted the interest of a theoretical study because of its low‐lying electronically degenerate excited states, which are prone to Jahn–Teller (JT) effects.^[18]^

We carried out spectroscopic investigations on molecular NiF_3_ and NiF_4_ for the first time. Different methods were applied to produce these species which were subsequently isolated in cryogenic rare‐gas matrices. First, nickel atoms were vaporized by heating a nickel wire and allowed to react with elemental fluorine diluted in argon prior to deposition on a matrix support at 10 K. Vaporization of atomic nickel was also achieved by laser ablation using the 1064 nm fundamental of a Nd:YAG laser focused onto a rotating nickel target and trapping the products at 6–15 K. The laser ablation process is associated with a hot plasma plume and a bright broad‐band radiation, where excited nickel atoms can react with elemental fluorine and atomic fluorine radicals seeded in excess of noble gases (neon or argon). This method is particularly useful for the generation of highly fluorinated species.[^13^, ^19^] For example, laser ablation from a metallic cobalt target in a fluorine/argon gas mixture clearly produces not only molecular CoF, CoF_2_ and CoF_3_ but also CoF_4_ (for experimental details see the Supporting Information), which could be detected using its known IR band in solid argon reported in a previous study,^[12]^ in which CoF_4_ was obtained at 650 K from solid mixtures of CoF_3_ and TbF_4_ as atomic fluorine source in a perfluorinated nickel effusion cell.^[12]^ The wavenumber ordering of the ν_Co‐F_ modes is CoF_4_ > CoF_3_ > CoF_2_ ≫ CoF, see the Supporting Information.

Thermally evaporated Ni atoms react with F_2_ on deposition (Figure 1) to yield limited amounts of NiF_2_ at 779.4 cm^−1[20]^ and a broad band at 625.8 cm^−1^ assigned to molecular NiF on the basis of the gas‐phase IR band of ^58^NiF (^2^Π_3/2_) at 634.7 cm^−1^,^[21]^ and our computed value of 639.1 cm^−1^ at the CCSD(T)/AVT(Q)Z‐DK level (Table S4.2). UV and broadband photolysis, associated with the formation of F radicals, resulted in the dramatic growth of the bands due to NiF_2_ (Figure 1 (a,d)), and two additional sets of overlapping bands close to 800 cm^−1^. Like the NiF_2_ bands the well resolved ^58, 60^Ni isotopic pattern on the 800 cm^−1^ bands is consistent with linear NiF_2_ units (Table S3.1).^[22]^ These new bands were assigned to matrix sites of NiF_2_ in F_2_/Ar matrices (see the Supporting Information).

**Figure 1:**
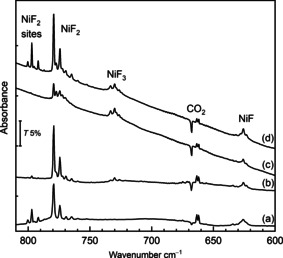
IR spectra of reaction products from thermally evaporated Ni atoms in 0.5 %F_2_/Ar matrices: a) after deposition and broadband photolysis, b) after annealing to 25 K, c) after deposition and annealing to 25 K, d) after broadband photolysis.

On annealing (Figure 1 (b,c)), the bands due to NiF_2_ increased while those close to 800 cm^−1^ decreased, and weak bands at 735–724 cm^−1^ appeared. These latter bands are assigned to the asymmetric stretch of molecular NiF_3_, trapped in different matrix sites. CCSD(T) calculations predict a ^4^A_2_ *′* ground state with *D*_3h_ structure for molecular NiF_3_ (Scheme 1, Table S6.3) and a single infrared active E*′* stretching fundamental about 40 cm^−1^ below that of NiF_2_ (Table S6.4).

**Scheme 1:**
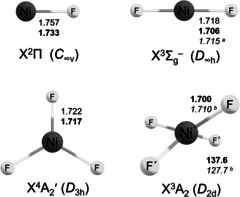
Structures of NiF_*n*_ (*n=*1–4) species. Values calculated at the RCCSD(T)/AVTZ(NREL) and the RCCSD(T)/AVTZ‐DK (in bold) levels of theory. [a] Experimental value from a gas phase electron diffraction study.^[23]^ [b] CASPT2/AVTZ‐DK.

Subsequent nickel laser ablation experiments fully corroborate the assignment of NiF_3_ in solid argon (Figure 2). Due to the formation of considerably larger amounts of atomic fluorine radicals in the laser ablation experiment, the yield of NiF_3_ increased and further rose by annealing the deposit to 20 K (Figure 2). The NiF_3_ region is even more congested in the laser ablation experiment due the presence of different matrix sites. UV radiation of *λ*=273 nm (LED) depletes the NiF_3_ bands (band points upwards in Figure S4b) and forms NiF_2_, while *λ*=193 nm laser radiation destroyed both, NiF_2_ and NiF_3_ (Figure S4d). More interestingly, in these experiments a new band at 749.1 cm^−1^ with a distinct Δν(^58/60^Ni) isotope splitting of 5.1 cm^−1^ occurred (Figure 2). This band was very difficult to detect in the thermal evaporation experiment due to its much lower intensity. The carrier of this band appears to be formed by fluorination of NiF_3_, since it can only be observed when a sufficient amount of NiF_3_ is available and its intensity grows along with that of the NiF_3_ band, i.e. by annealing and with higher fluorine concentrations of the F_2_/Ar mixture (Figure S5). Based on this behavior, the new band is assigned to the strongest IR band of molecular NiF_4_. For a tetrahedral tetrafluoride only a single infrared‐active Ni−F stretching mode is expected, however, for the *d* ^6^ electronic configuration of NiF_4_ a Jahn–Teller distorted *D*_2d_ structure is predicted at all levels of theory (Figure S7.1).

**Figure 2:**
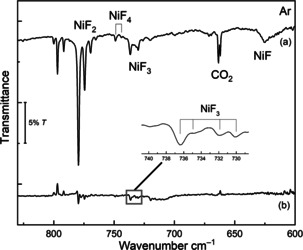
IR spectrum of the reaction products of laser‐ablated Ni with F_2_ (0.5 %) seeded in excess argon: a) co‐deposited for 60 min at 15 K, b) difference spectrum after annealing to 20 K.

We are not aware of previous high‐level ab initio calculations for molecular NiF_4_. We carried out quantum‐chemical investigations of various energetically low‐lying electronic states of NiF_4_, especially of the lowest‐energy triplet (^3^A_2_) and quintet (^5^B_1_, ^5^A_1_) states of *D*_2d_ symmetry (Table S3.4). For the triplet ground state (Scheme 1) a strong IR band of E symmetry is predicted for NiF_4_ about 11 cm^−1^ blue‐shifted from the E*′* band of NiF_3_ at the CCSD(T)/AVTZ‐DK level (Table 1), which is in excellent agreement with the observation. The much weaker predicted *B*_2_ band of NiF_4_ (Tables S7.2, S7.4, S7.6) was not detected. All four nickel fluorides are also observed in solid neon matrices (Tables 1, S3.3).

**Table 1:** Vibrational frequencies observed in laser‐ablation experiments and computed harmonic frequencies (in cm^−1^) for NiF_*n*_ (*n=*1–4).

Species	Ground State	Sym.	calcd^[a]^			Exp. (Ne Matrix)		Exp. (Ar Matrix)	
				^58^Ni	^60^Ni	^58^Ni	^60^Ni	^58^Ni	^60^Ni
NiF	^2^Π	*C*_∞v_	NR^[b]^	612.98 (Σ^+^)	610.46	646.2	643.5	625.8 (broad)	
			DK^[b]^	639.13 (Σ^+^)	636.50				
NiF_2_	^3^Σ_g_^−^	*D*_∞h_	NR^[c]^	806.42 (Σ_u_^+^)	801.09	800.1	794.9	779.5	774.4
			DK^[c]^	819.17 (Σ_u_^+^)	813.75				
			NR^[b]^	810.25 (Σ_u_^+^)	804.89				
			DK^[b]^	824.62 (Σ_u_^+^)	819.17				
NiF_3_	^4^A_2_ *′*	*D*_3h_	NR^[c]^	762.95 (E*′*)	758.67	743.8	740.0	736.5	731.7
			DK^[c]^	767.83 (E*′*)	763.54				
NiF_4_	^3^A_2_	*D*_2d_	NR^[c]^	767.24 (E)	762.56	762.3	757.2	749.1	744.0
			DK^[c]^	778.50 (E)	773.71				

[a] Non‐relativistic (NR) and with scalar‐relativistic Douglas‐Kroll‐Hess (DK) Hamiltonian; [b] RCCSD(T)/AVQZ; [c] RCCSD(T)/AVTZ.

A comparison of experimental M−F stretching bands of high‐valent first‐row metal fluorides is particularly revealing. The molecular trifluorides of M = Fe, Co, and Ni all adopt *D*_3h_ structures, and their stretching fundamentals were found in Ne matrices, in a very narrow range, at 743.6 cm^−1^ (Fe),^[13]^ 748.2 cm^−1^ (Co), and 743.8 cm^−1^ (Ni, Table 1). For a series of molecules with similar structures one expects a strong correlation between stretching frequencies and bond lengths, a trend that also applies to the bond length of CoF_3_ (1.722 Å) and NiF_3_ (1.722 Å, Table S3.5) obtained at the CCSD(T)/AVTZ level. A similar frequency‐bond length correlation (Table S3.5) is expected for the stretching vibrations of the tetrafluorides MF_4_ of Fe to Ni observed in solid argon, which also appear in a narrow spectral range (757 cm^−1^ (M = Fe),^[13]^ 767.8 (Co),^[12]^ 749.1 (Ni)). However, this correlation is less accurate due to the Jahn–Teller deformed *D*_2d_ molecular structures of FeF_4_ and NiF_4_ and due to stronger matrix interactions in solid argon.

Apart from these similarities, the higher nickel fluorides are strikingly different from their third‐row predecessors. The rule of thumb that higher fluorides have shorter and stronger M−F bonds that applies to ionic metal fluorides does not apply to the higher nickel fluorides. While CCSD(T)/AVTZ calculations predict a successive M−F bond shortening for the ionic iron fluorides FeF_*n*_, with *n=*1–4,^[13]^ and, albeit significantly weakened, also for CoF_*n*_, *n=*2–4, the bond length in NiF_*n*_, *n=*2–4, remains almost unchanged (Table S3.5). This different trend for nickel and its predecessors can be traced back to fundamental aspects of their atomic structures, such as a higher nickel ionization energy. This leads to a considerable covalent character of the Ni−F bonds, associated with a low‐spin triplet electronic ground‐state of NiF_4_ and a considerable radical character of the fluorine ligands (Figure S11).[^7^, ^8^]

From these experiments we conclude that NiF_2_, initially formed from the reaction of nickel atoms and elemental fluorine, can be regarded as chemically inert to elemental fluorine, but reacts rapidly with atomic fluorine radicals to form NiF_3_ and further to NiF_4_. It has also been shown that the reaction of Ni atoms with elemental F_2_ to produce NiF_2_ requires UV photolysis to yield appreciable quantities of product under the cryogenic conditions applied here. Given the highly exothermic reaction energy of this reaction (Table 2), this observation indicates a considerable reaction barrier and the necessity for fluorine radicals.

**Table 2:** Calculated thermochemistry of nickel fluorides in kJ mol^−1^.

	Δ*E°* (kJ mol^−1^), CCSD(T)/AVTZ‐DK
Ni + F → NiF	−426.16
NiF + F → NiF_2_	−507.44
NiF + F_2_ → NiF_3_	−517.91
NiF_2_ + F → NiF_3_	−163.50
NiF_3_ + F → NiF_4_	−76.04

To summarize, molecular NiF_4_ (*D*_2d_) and NiF_3_ (*D*_3h_) were produced, isolated in solid noble‐gas matrices, and spectroscopically identified aided by quantum‐chemical calculations. Their weak Ni−F bonds and a considerable fluorine radical character make these molecular species very powerful fluorination and oxidation agents.

## Conflict of interest

The authors declare no conflict of interest.

## Supporting information

As a service to our authors and readers, this journal provides supporting information supplied by the authors. Such materials are peer reviewed and may be re‐organized for online delivery, but are not copy‐edited or typeset. Technical support issues arising from supporting information (other than missing files) should be addressed to the authors.

Supplementary
