# Supplementary material for: Searching for Monomeric Nickel Tetrafluoride: Unravelling Infrared Matrix Isolation Spectra of Higher Nickel Fluorides
Source: Angew Chem Int Ed Engl. 2021 Jan 28;60(12):6391–4. doi: 10.1002/anie.202015501 (PMC7986428; doi:10.1002/anie.202015501)
Supplement: Supplementary file 1 — Supplementary [file ANIE-60-6391-s001.pdf]

Angewandte Chemie
Eine Zeitschrift der Gesellschaft Deutscher Chemiker

Supporting Information

Searching for Monomeric Nickel Tetrafluoride: Unravelling Infrared Matrix Isolation Spectra of Higher Nickel Fluorides

Lin Li, Ahmed K. Sakr, Tobias Schlöder, Siri Klein, Helmut Beckers, Marios-Petros Kitsaras, Howard V. Snelling, Nigel A. Young, Dirk Andrae, and Sebastian Riedel**

anie_202015501_sm_miscellaneous_information.pdf

Contents

Part 1. Experimental and Computational Details and Details about Spectral Assignments and Reaction Mechanism.

1.1. Experimental and computational details 3

1.2. Computational details 3

1.3. Reaction of laser ablated cobalt atoms with fluorine 4

1.4. Assignment of binary nickel fluorides isolated in solid rare-gas matrices 4

1.5. Electronic absorption spectra 5

1.6. Proposed reaction mechanism 6

1.7. References to part 1 6

Part 2. Supporting Figures

Spectra (Figures S1-S10) and spin densities of NiF_n (n = 1-4, Figure S11) 8

Part 3. Supporting Tables

3.1. Experimental vibrational frequencies of nickel fluorides (in cm^{-1}) observed on thermal evaporation of nickel in the presence of elemental fluorine. (Table S3.1) 19

3.2. Electronic absorptions (cm^{-1}) and assignments for Ni atoms isolated in Ar matrix. (Table S3.2) 19

3.3. Experimental vibrational frequencies of nickel fluorides (in cm^{-1}) observed by laser ablation of nickel in the presence of elemental fluorine. (Table S3.3) 20

3.4. Electronic states, structural parameters (Å, deg) and electronic energy differences (kJ mol^{-1}) of selected states of NiF_3 and NiF_4 (Table S3.4) 20

3.5. Computed M−F bond length [Å] and comparison of computed and experimental vibrational frequencies [cm^{-1}] of late TM fluorides MF_n (M = Fe, Co, Ni and Cu; n = 2-4) (Table S3.5) 21

3.6. References to part 3 21

Part 4. CASSCF, MRCI, MRCI+Q and CCSD(T) calculations on NiF ($^2\Pi$) (Tables S4.1-S4.2) 22

Part 5. RHF, MP2, CISD and CCSD(T) calculations on NiF_2 ($^3\Sigma_g^-$, $^1\Sigma_g^+$)
(Tables S5.1-S5.8) 24

Part 6. RHF, MRCI and CCSD(T) calculations on NiF_3 ($^2A_1$ (C_{2v}) and $^4A_2'$ (D_{3h})) (Tables S6.1-S6.4) 32

Part 7. NiF_4

Description of a D_{2d}-distorted NiF_4 tetrahedron (Figure S7.1) 36

DFT calculation on NiF_4 ($^3A_2$ and $^5B_1$) (Tables S7.1-S7.2) 37

CCSD(T) and CASPT2 calculations on NiF_4 ($^3A_2$, $^5A_1$ and $^5B_1$)
(Tables S7.3-S7.9) 38

Part 1. Experimental and Computational Details and Details about Spectral Assignments and Reaction Mechanism.

1.1. Experimental details

Matrix samples were prepared by co-deposition of laser-ablated excited nickel atoms or NiF_2 molecules with different concentration diluted F_2 in neon (99.999%, Air Liquide) and argon (99.999%, Sauerstoffwerk Friedrichshafen). The bulk NiF_2 target was prepared in a hydraulic lab press and mounted on a target holder. The gases were mixed in a custom-made stainless steel mixing chamber equipped with a manometer. The stainless steel F_2 storage cylinder was cooled in liquid nitrogen to avoid impurities. The mixing chamber was connected to the matrix chamber by a stainless-steel capillary. The reactants were condensed onto a gold mirror cooled to 6 K (neon) and 5-15 K (argon) using a closed-cycle helium cryostat (Sumitomo Heavy Industries, RDK205D) inside the matrix chamber.[1] For the laser ablation of targets, the 1064 nm fundamental of a Nd:YAG laser (Continuum, Minilite II, repetition rate: 10 Hz, pulse width: 10 ns, pulse energy up to 40 mJ) was focused onto the rotating target through a hole in the gold mirror. Selective radiations of the matrix were used λ = 617, 470, 455, 273 nm (LEDs) and 266 nm (laser), respectively. Another radiation of excimer laser λ = 193 nm was also used. Matrices were annealed to different temperatures, as well as using a mercury lamp (Osram HQL 250) cooperating with different wavelength edge filters. Infrared (IR) spectra were recorded on a Bruker Vertex 80 FT-IR spectroscopy with 0.5 cm^{-1} resolution in the region between 4000-450 cm^{-1} using a liquid-nitrogen cooled MCT detector.

The thermal evaporation studies (performed at the University of Hull) used five strands of 0.5 mm nickel wire (99.9% Aldrich) tightly wound together and made into a coiled filament and heated using *ca*. 30 A at 1 V. To avoid heating of the window and matrix deposit, and to limit the reaction of the fluorine with the heated filament, a copper disc with a 5 mm aperture was placed between the furnace and the vacuum chamber containing the deposition window. Details of the matrix-isolation setup used in these experiments are published elsewhere.[2] The F_2/Ar mixtures were prepared using standard manometric procedures from 10% F_2/Ar (Air Liquide) and Ar (99.999% Energas) using a metal vacuum line. The vacuum line, reservoirs and vacuum chambers were well passivated. The reactants were condensed onto a CsI (IR) or CaF_2 (UV-vis-NIR) window held at *ca*. 10 K by an APD DE-204 cryostat. Matrices were annealed to different temperatures using a Scientific Instruments SI 9600-1 digital controller and silicon diodes. Broadband photolysis (λ > 250 nm) was carried with a LOT-Oriel 200 W Hg(Xe) lamp, which was also used with 400-700 nm and 200-410 nm filters. IR spectra were recorded using a KBr beam splitter and DTGS detector on a Bruker EquNox55 FTIR instrument. Separate electronic absorption spectra were recorded on a Varian Cary 5E UV-vis-NIR spectrometer.

1.2. Computational Details

The MOLPRO program19 package[3] was used for quantum-chemical ab-initio calculations on several neutral nickel fluoride species. Scalar relativistic all-electron calculations were performed using the second-order Douglas-Kroll-Hess (DK) Hamiltonian[4] and corresponding (augmented) correlation-consistent polarized valence n-tuple Gaussian basis sets, denoted as (aug-)cc-pVnZ-DK and abbreviated as (A)VnZ-DK (n = D, T, Q).[5] In all tables, the basis set label suffix -DK always implies the use of the DK Hamiltonian. Calculations were done at different levels of theory up to coupled-cluster (CC) level. For each molecule, several possible spin states of all possible spatial symmetries (irreducible representations within a chosen point group) were initially considered by running state-averaged CASSCF calculations. Candidates for the molecular electronic ground state were thus identified and further studied. In every single case, the most important single configuration found at the CASSCF level was selected as reference for subsequent RHF-RCCSD(T) calculations, in order to obtain a fully optimized molecular structure for the chosen electronic state. Normal mode analysis in harmonic

approximation was done to confirm that the optimized structure represents a minimum on the potential energy hypersurface. Selected results from the quantum-chemical calculations are presented below.

In order to calculate the relative IR intensities of the vibrational modes of NiF_4, additional calculations at density functional theory (DFT) level were carried for this molecule using the Gaussian16 program package and the B3LYP functional as implemented therein.[6] All DFT calculations were performed within the restrictions of the D_{2d} point group.

1.3. Reaction of Laser Ablated Cobalt Atoms with Fluorine

The IR spectrum obtained from laser ablation of metallic cobalt in a fluorine/argon gas mixtures (Figure S6), shows in addition to molecular CoF ($^3\Phi_i$, 637.8 cm^{-1}; in gas phase 662.6 cm^{-1} [7]), CoF_2 ($^4\Delta_g$, ν_3-band at 722.5 cm^{-1}) [8] the higher fluorides CoF_3 ($^5A_1'$, 736.9 cm^{-1} with matrix sites at 739.4 and 733.3 cm^{-1}) [9] and CoF_4 ($^6A_1$, medium band at 767.8 cm^{-1}). Their band positions agree very well with those from previous studies, [8–10] in which CoF_3 vapour was investigated at 800 K and CoF_4 was obtained at 650 K from solid mixtures of CoF_3 and TbF_4 as atomic fluorine source in a perfluorinated nickel effusion cell.[8] Thermal evaporation experiments also contained bands due to CoF (637.9 cm^{-1}), CoF_2 (722.8 cm^{-1}) and a collection of bands at 740.5, 737.1 and 733.2 cm^{-1} due to CoF_3 in a variety of sites, but with no evidence for the CoF_4 band at 767.8 cm^{-1}.

1.4. Assignment of Binary Nickel Fluorides Isolated in Solid Rare-Gas Matrices

In the solid argon deposit obtained from thermally evaporated nickel atoms and elemental fluorine the antisymmetric stretching vibration of molecular NiF_2 [11,12] appeared at 779.5 cm^{-1} for the ^{58}Ni isotopomer (Figures S1, S2, S3). Band positions for the $^{58/60/62}$Ni isotopes are well resolved for this vibrational mode (Table S3.1) from which a bond angle of the argon matrix-isolated NiF_2 molecule is estimated to 165° using a simple valence force field (SVFF),[13] compared to 165°, 154° and 152° reported previously.[8,12,14] However, for bond angles close to linearity a difference of only *ca.* 0.1 cm^{-1} in the isotope shifts already results in a change in bond angles from 180° to 160°,[15] it can be concluded that this estimate for argon-matrix isolated NiF_2 is consistent with a linear structure.[16]

Two set of bands with the highest wavenumber band at 800.2 cm^{-1} and a distinct Ni isotope splitting deserves special attention. Their $\Delta\nu$($^{58/60}$Ni) isotope splitting is close to that of molecular NiF_2 ($\Delta\nu$ = 5.1 cm^{-1}, Table S3.1, Figure S2), and the bond angle estimates from the isotope splitting (163 and 160°, respectively) indicates also for these bands a carrier with an almost linear F–Ni–F unit. It is tempting to assign these features at 800 cm^{-1} to a higher nickel fluoride such as NiF_4, since higher fluorides can be assumed to have higher frequency Ni–F stretching modes. However, quantum-chemically predicted ground-state structures of molecular NiF_4 (see Part 7) are inconsistent with the experimental observation of linear F–Ni–F units and with vibrational modes higher in frequency than NiF_2. We therefore conclude that the features close to 800 cm^{-1} are due to matrix sites of NiF_2 in F_2/Ar matrices. It should be noted that these sites are not formed when NiF_2 is thermally evaporated and trapped in solid argon,[11,12] or by photolysis of argon-matrix isolated NiF_2, but they are present when NiF_2 is generated by photolysis from Ni atoms and F_2, indicating that fluorine is probably present in the trapping site. Similar site effects for other difluorides have been observed previously.[1,17]

The bands observed in the region 736-729 cm^{-1} (Figures S1, S2, S3) grow on annealing on the expense of the NiF_2 matrix sites, remain constant under broadband photolysis (see difference spectra in Figure S1), and appear at relatively low annealing temperatures of 15 K. Since formation of dimers by annealing to 15 K would be unusual and the isotopic spacing observed for these bands is consistent with *ca.* 120°

bond angle, these bands were finally assigned in good agreement with our CCSD(T) calculation (Table S6.4) to the antisymmetric NiF_3 stretching vibration of molecular NiF_3 (D_{3h}). At least two different matrix-sites were observed for this species in these experiments, one with a main peak at 733.2 cm^{-1} and a pair at 730.1 and 726.1 cm^{-1}.

In further experiments studying the reaction of IR-laser ablated nickel atoms with elemental fluorine all four nickel fluorides NiF_n, n = 1-4, were also observed in solid neon matrices (Figure S7). As expected, their Ni−F stretching bands revealed a significant blue-shift compared to the argon matrix (Table S3.3). The antisymmetric NiF_3 stretch shows at least two well separated Ne-matrix sites, which are strongly depleted by selective λ = 266 nm laser radiation with formation of NiF_2 (Figure S7c). The photodecomposition of NiF_3 is associated with the depletion of only a single IR band in the Ni−F stretching region which clearly supports its quantum-chemically predicted D_{3h} structure. Another general feature of metal-laser-ablation and Ne-matrix isolation is the formation of the polyfluorine monoanions $[F_3]^-$ and $[F_5]^-$.[18] Their characteristic bands are indicated in Figure S7. We note that the laser-ablation process is associated with a hot plasma plume and a bright broad-band radiation. In these experiments there is photolysis all the time and the high wavenumber matrix sites of NiF_2 are always present. We also studied the reaction of laser-ablated NiF_2 molecules with elemental fluorine using fluorine/neon mixtures and a fluorine/argon mixtures. These experiments produced two remarkable results. We first noticed unprecedented high intensities of the E′ stretching vibration of NiF_3 that were achieved despite the low fluorine content of the gas mixture (0.05 % F_2 in Ne) used in these experiments (Figure S8). The second observation is that the high-wavenumber matrix-site of NiF_2 in solid argon were not observed in these experiments. From this observation it is assumed that these high-wavenumber features are likely formed by UV photolysis from Ni atoms and F_2, which are both present in the solid matrices.

1.5. Electronic Absorption spectra

The electronic absorption spectra of Ni atoms in rare gas matrices have been well studied. [19–32] In the original reports [19–21] it was assumed that the atomic $^3F_4$ ($3d^84s^2$) ground state was also the ground state of the matrix isolated atoms. However, later work [22–32] showed that there were both $^3F_4$ and $^3D_3$ ($3d^94s^1$) states within Ar, Kr and Xe matrices, but only $^3F_4$ in solid Ne. In the gas phase the $^3D_3$ excited state is *ca.* 205 cm^{-1} above the $^3F_4$ ground state, and it was suggested that the inversion was the result of greater matrix induced repulsion of the 4*s* compared to the 3*d* electrons. Our spectra of thermally produced Ni atoms in argon matrices are shown in Figure S9 and they are in very good agreement with the earlier reports. [19–32] On deposition at *ca.* 10 K there are features due to both the $^3D_3$ state and the $^3F_4$ state as given in Table S3.2, but no evidence for dimers and trimers which have features in the range 18900-27000 cm^{-1} [21] On broadband photolysis the intensity of the $^3D_3$ features at 29000-38000 cm^{-1} and 45500-50000 cm^{-1} decreased, whilst the $^3F_4$ features at 42000-45500 cm^{-1} increased markedly. Similar photolysis behaviour has been observed for Ni atoms in solid Kr, although the $^3F_4$ features were initially assigned to dimers [24] before subsequent work identified them to be due to $^3F_4$ [30] and that the photolysis involved z-type $^3P_2^0 \leftarrow {}^3D_3$ excitation, followed by decay to the $^3F_4$ ground state. This photolysis mechanism was also supported by Vala et al. [26] On annealing, the features associated with the $^3F_4$ state decay much more quickly than those of the $^3D_3$ state, which has also been observed previously. [25] Although the photolysis and annealing behaviours have been identified previously, the data in Figure S9 is the first time both have been presented for argon matrices, and are necessary to follow the changes in Figure S10, where F_2 has been introduced into the matrix.

In the presence of F_2 (Figure S10), there is the characteristic spectrum of Ni atoms in both $^3F_4$ and $^3D_3$ states on deposition. After broadband photolysis there is a dramatic reduction in the intensity of the features associated with the $^3D_3$ state, but a less marked reduction in the $^3F_4$ bands. This is in contrast to pure argon where there was a moderate transfer of intensity from the $^3D_3$ to $^3F_4$ features. On annealing, the remaining $^3D_3$ bands reduce slightly, but the $^3F_4$ peaks decay quite markedly as observed in pure argon. There are also two new broad bands at 45660 and 48950 cm^{-1} not observed in the pure argon

spectra, which slightly increase in intensity after annealing to 15 and 20 K, followed by a slight decrease on annealing to 25 and 30 K. Since the 45660 and 48950 cm^{-1} bands are only observed in the presence of fluorine, this indicates that they belong to a nickel fluoride species. In previous studies, no UV absorptions were observed for NiF_2, but this was carried out with photographic plates.[14] For $NiCl_2$ in argon matrices an intense charge transfer absorption has been observed at 28350 cm^{-1}.[16] Using an optical electronegativity value of 3.0 for χ_{Cl}, allows for an estimate of χ_{Ni} of 2.1 in triatomics such as $NiCl_2$ and NiF_2, which is in good agreement with values of 2.0-2.1 reported for tetrahedral Ni(II).[33] This value of χ_{Ni} in combination with a χ_F value of 3.9 predicts charge transfer transitions of *ca*. 55000 cm^{-1} for NiF_2.[33] For higher oxidation state nickel fluorides, the lowest energy charge transfer transition would be expected at significantly lower energies. Therefore, it is reasonable to assign the bands at 45660 and 48950 cm^{-1} to NiF_2 charge transfer transitions, which have not been reported previously.

The observation of high wavenumber "sites" of NiF_2 located in the IR spectra in the region from 800-791 cm^{-1}, which appeared after photolysis of solid argon matrices containing nickel atoms and F_2 (Figures S1, S2, S3), but not by photolysis of argon matrices containing NiF_2 and F_2, lets us assume, that these less stable matrix sites are likely formed from excited nickel atoms and F_2 after photo-excitation. Once formed, the less stable site species then decay to the stable NiF_2 site. Further detailed studies to support this preliminary assumption are necessary, but are not the subject of this work.

1.6. Proposed Reaction Mechanism

From the matrix-isolation experiments we conclude that the reactivity of molecular NiF_2 is markedly different from that of CoF_2. As shown in Figure S6, the amount of CoF_4 produced in the laser-ablation process can be considerably higher than that of CoF_3. This observation suggests that initially formed CoF_2 (Eq. **1**) further reacts with F_2 to yield CoF_4 (Eq. **3**), while CoF_3 is formed either by decomposition of CoF_4 or by the reaction of CoF_2 and fluorine radicals (**2**). Interestingly, the relative yield of CoF_4 strongly depends on the temperature of the matrix support (Figure S6): At slightly higher deposition temperatures significantly higher amounts of CoF_4 were obtained, indicating that reaction (**3**) likely takes place during the deposition in the "condensed" phase prior to the complete confinement of the reaction products in the cryogenic matrix. To the contrary, initially formed NiF_2 (Eq. **4**) can be regarded as chemically inert to elemental fluorine, but react rapidly with atomic fluorine radicals to form NiF_3 (Eq. **5**) and further to NiF_4 (**6**). It has also been shown that the reaction of Ni atoms with elemental F_2 to produce NiF_2 (Eq. **4**) requires UV photolysis to yield appreciable quantities of product under the cryogenic conditions applied here. Given the highly exothermic reaction energy of this reaction (Table 2, main text), this observation indicates a considerable reaction barrier and the necessity for fluorine radicals.

$Co(g) + F_2(g) \rightarrow CoF_2(g)$ (**1**)
$CoF_2(g) + F(g) \rightarrow CoF_3(g)$ (**2**)
$CoF_2(g) + F_2(g) \rightarrow CoF_4(g)$ (**3**)

$Ni(g) + F_2(g) \rightarrow NiF_2(g)$ (**4**)
$NiF_2(g) + F(g) \rightarrow NiF_3(g)$ (**5**)
$NiF_3(g) + F(g) \rightarrow NiF_4(g)$ (**6**)

1.7. References to Part 1

[1] A. V. Wilson, T. Nguyen, F. Brosi, X. Wang, L. Andrews, S. Riedel, A. J. Bridgeman, N. A. Young, *Inorg. Chem.* **2016**, *55*, 1108.
[2] J. F. Rooms, A. V. Wilson, I. Harvey, A. J. Bridgeman, N. A. Young, *Phys. Chem. Chem. Phys.* **2008**, *10*, 4594.

[3] H.-J. Werner, P. J. Knowles, G. Knizia, F. R. Manby, M. Schütz, P. Celani, W. Györffy, D. Kats, T. Korona, R. Lindh, A. Mitrushenkov, G. Rauhut, K. R. Shamasundar, T. B. Adler, R. D. Amos, S. J. Bennie, A. Bernhardsson, A. Berning, D. L. Cooper, M. J. O. Deegan, A. J. Dobbyn, F. Eckert, E. Goll, C. Hampel, A. Hesselmann, G. Hetzer, T. Hrenar, G. Jansen, C. Köppl, S. J. R. Lee, Y. Liu, A. W. Lloyd, Q. Ma, R. A. Mata, A. J. May, S. J. McNicholas, W. Meyer, T. F. Miller III, M. E. Mura, A. Nicklass, D. P. O'Neill, P. Palmieri, D. Peng, K. Pflüger, R. Pitzer, M. Reiher, T. Shiozaki, H. Stoll, A. J. Stone, R. Tarroni, T. Thorsteinsson, M. Wang, M. Welborn, *MOLPRO, version 2019.2, a package of ab initio programs*.
[4] a) M. Douglas, N. M. Kroll, *Ann. Phys.* **1974**, *82*, 89; b) B. A. Hess, *Phys. Chem. A* **1985**, *32*, 756.
[5] N. B. Balabanov, K. A. Peterson, *J. Chem. Phys.* **2005**, *123*, 64107.
[6] a) M. J. Frisch, G. W. Trucks, H. B. Schlegel, G. E. Scuseria, M. A. Robb, J. R. Cheeseman, G. Scalmani, V. Barone, G. A. Petersson, H. Nakatsuji, X. Li, M. Caricato, A. V. Marenich, J. Bloino, B. G. Janesko, R. Gomperts, B. Mennucci, H. P. Hratchian, J. V. Ortiz, A. F. Izmaylov, J. L. Sonnenberg, D. Williams-Young, F. Ding, F. Lipparini, F. Egidi, J. Goings, B. Peng, A. Petrone, T. Henderson, D. Ranasinghe, V. G. Zakrzewski, J. Gao, N. Rega, G. Zheng, W. Liang, M. Hada, M. Ehara, K. Toyota, R. Fukuda, J. Hasegawa, M. Ishida, T. Nakajima, Y. Honda, O. Kitao, H. Nakai, T. Vreven, K. Throssell, J. A. Montgomery, Jr., J. E. Peralta, F. Ogliaro, M. J. Bearpark, J. J. Heyd, E. N. Brothers, K. N. Kudin, V. N. Staroverov, T. A. Keith, R. Kobayashi, J. Normand, K. Raghavachari, A. P. Rendell, J. C. Burant, S. S. Iyengar, J. Tomasi, M. Cossi, J. M. Millam, M. Klene, C. Adamo, R. Cammi, J. W. Ochterski, R. L. Martin, K. Morokuma, O. Farkas, J. B. Foresman, and D. J. Fox, *Gaussian 16*, Gaussian, Inc., Wallingford CT, **2016**; b) A. D. Becke, *Phys. Rev. A* **1988**, *38*, 3098; c) C. Lee, W. Yang, R. G. Parr, *Phys. Rev. B* **1988**, *37*, 785; d) A. D. Becke, *J. Chem. Phys.* **1993**, *98*, 5648.
[7] a) A. G. Adam, L. P. Fraser, W. D. Hamilton, M. C. Steeves, *Chem. Phys. Lett.* **1994**, *230*, 82; b) R. S. Ram, P. F. Bernath, S. P. Davis, *J. Mol. Spectrosc.* **1995**, 158.
[8] J. W. Hastie, R. H. Hauge, J. L. Margrave, *J. Chem. Soc. Comm.* **1969**, 1452.
[9] V. N. Bukhmarina, A. Y. Gerasimov, Y. B. Predtechenskii, *Vib. Spectrosc.* **1992**, *4*, 91.
[10] J. V. Rau, S. Nunziante Cesaro, N. S. Chilingarov, G. Balducci, *Inorg. Chem.* **1999**, *38*, 5695.
[11] D. A. van Leirsburg, C. W. DeKock, *J. Phys. Chem.* **1974**, *78*, 134.
[12] J. W. Hastie, R. H. Hauge, J. L. Margave, *High Temp. Sci.* **1969**, *1*, 76.
[13] K. Nakamoto, *Infrared and Raman spectra of inorganic and coordination compounds A: Theory and Applications in Inorganic Chemistry*, John Wiley & Sons Inc, Hoboken, N.J., **2009**.
[14] D. E. Milligan, M. E. Jacox, J. D. McKinley, *J. Chem. Phys.* **1965**, *42*, 902.
[15] a) I. R. Beattie, P. J. Jones, N. A. Young, *Chem. Phys. Lett.* **1991**, *177*, 579; b) S. H. Ashworth, F. J. Grieman, J. M. Brown, P. J. Jones, I. R. Beattie, *J. Am. Chem. Soc.* **1993**, *115*, 2978.
[16] O. M. Wilkin, N. Harris, J. F. Rooms, E. L. Dixon, A. J. Bridgeman, N. A. Young, *J. Phys. Chem. A* **2018**, *122*, 1994.
[17] A. V. Wilson, A. J. Roberts, N. A. Young, *Angew. Chem. Int. Ed.* **2008**, *47*, 1774.
[18] a) F. A. Redeker, H. Beckers, S. Riedel, *RSC Adv.* **2015**, *5*, 106568; b) T. Vent-Schmidt, F. Brosi, J. Metzger, T. Schlöder, X. Wang, L. Andrews, C. Müller, H. Beckers, S. Riedel, *Angew. Chem. Int. Ed.* **2015**, *54*, 8279; c) F. Brosi, T. Vent-Schmidt, S. Kieninger, T. Schlöder, H. Beckers, S. Riedel, *Chem. Eur. J.* **2015**, *21*, 16455.
[19] W. Klotzbuecher, G. A. Ozin, *Inorg. Chem.* **1976**, *15*, 292.
[20] D. M. Mann, H. P. Broida, *J. Chem. Phys.* **1971**, *55*, 84.
[21] M. Moskovits, J. E. Hulse, *J. Chem. Phys.* **1977**, *66*, 3988.
[22] K. Jacobi, D. Schmeisser, D. M. Kolb, *Chem. Phys. Lett.* **1980**, *69*, 113.
[23] B. Breithaupt, J. E. Hulse, D. M. Kolb, H. H. Rotermund, W. Schroeder, W. Schrittenlacher, *Chem. Phys. Lett.* **1983**, *95*, 513.
[24] R. Grinter, D. R. Stern, *J. Chem. Soc., Faraday Trans. 2* **1983**, *79*, 1011.
[25] W. Schroeder, R. Grinter, W. Schrittenlacher, H. H. Rotermund, D. M. Kolb, *J. Chem. Phys.* **1985**, *82*, 1623.
[26] M. Vala, M. Eyring, J. Pyka, J.‐C. Rivoal, C. Grisolia, *J. Chem. Phys.* **1985**, *83*, 969.
[27] M. Tomonari, H. Tatewaki, T. Nakamura, *J. Chem. Phys.* **1986**, *85*, 2875.
[28] M. Rasanen, L. A. Heimbrook, V. E. Bondybey, *J. Mol. Struct.* **1987**, *157*, 129.
[29] G. H. Jeong, K. J. Klabunde, *J. Chem. Phys.* **1989**, *91*, 1958.
[30] C. P. Barrett, R. G. Graham, R. Grinter, *Chem. Phys.* **1984**, *86*, 199.
[31] T. A. Cellucci, E. R. Nixon, *J. Chem. Phys.* **1984**, *81*, 1174.
[32] W. Schrittenlacher, W. Schroeder, H. H. Rotermund, D. M. Kolb, *Chem. Phys. Lett.* **1984**, *109*, 7.
[33] Lever. A. B. P. (Ed.) *Inorganic Electronic Spectroscopy (II)*, Elsevier, **1984**.

Part 2: Supporting Figures

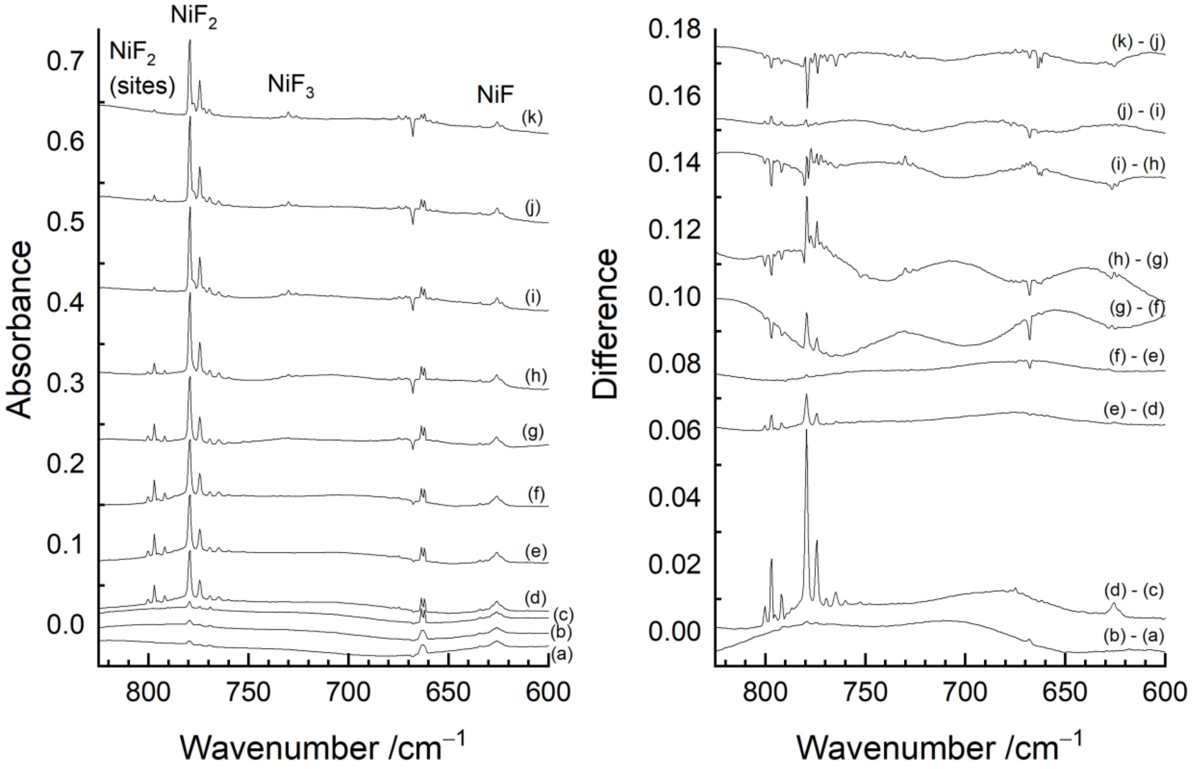

Figure S1. IR spectra (left) and difference spectra (right) of the reaction products of thermally evaporated Ni atoms in 0.5% F_2/ Ar matrix.

(a) After deposition (2 cm^{-1} resolution)
(b) After 10 min visible photolysis (2 cm^{-1} resolution)
(c) After 10 min visible photolysis (1 cm^{-1} resolution)
(d) After 10 min 410-200 nm photolysis (1 cm^{-1} resolution)
(e) After 10 min broadband photolysis (1 cm^{-1} resolution)
(f) After 10 visible photolysis (1 cm^{-1} resolution)
(g) After 5 min annealing at 15 K (1 cm^{-1} resolution)
(h) After 5 min annealing at 20 K (1 cm^{-1} resolution)
(i) After 5 min annealing at 25 K (1 cm^{-1} resolution)
(j) After 10 min broadband photolysis (1 cm^{-1} resolution)
(k) After 5 min annealing to 30 K (1 cm^{-1} resolution)

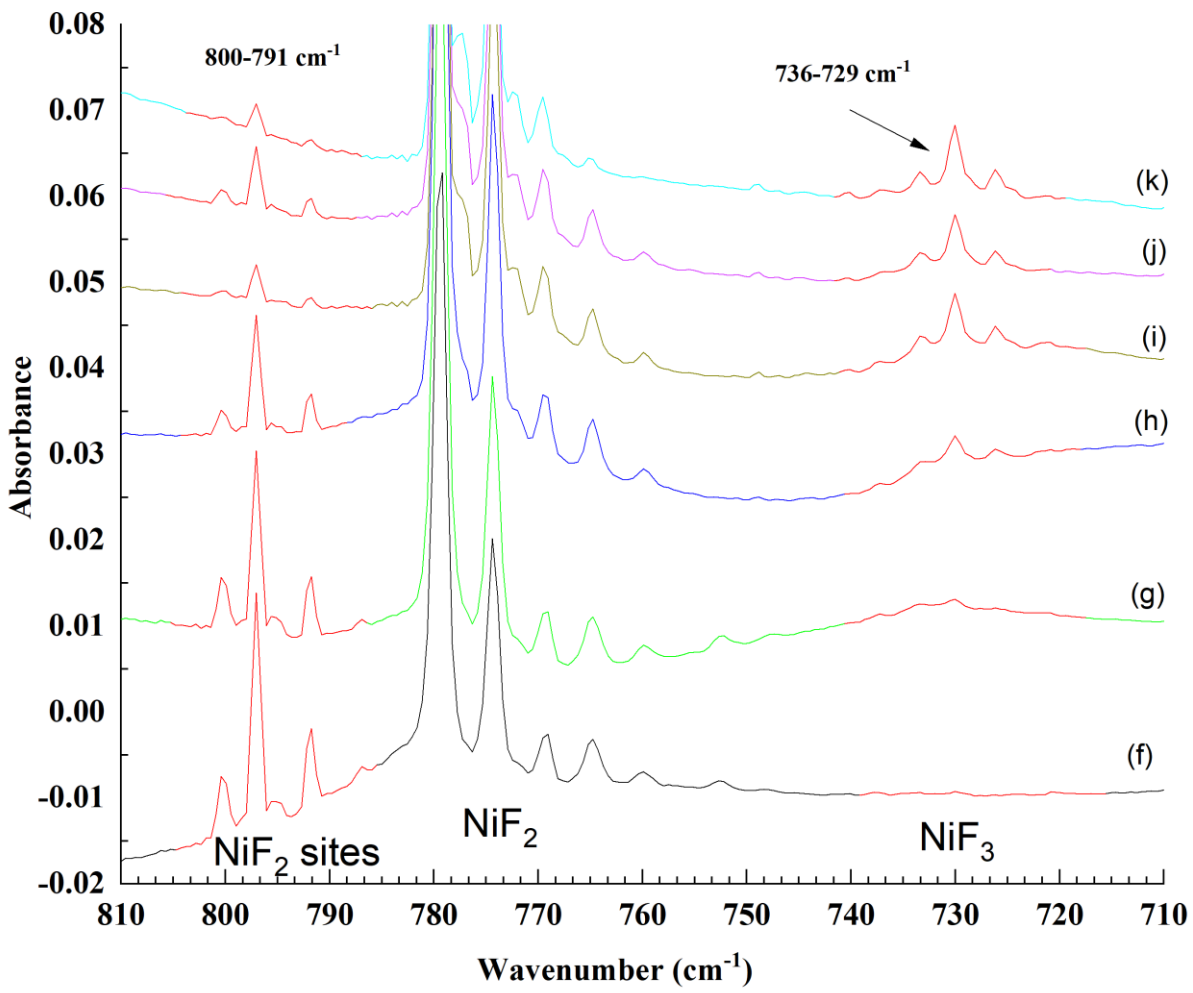

Figure S2. Expansion of IR spectra of the reaction products of thermally evaporated Ni atoms in 0.5% F_2/ Ar matrix.

(f) After photolysis sequence (1 cm^{-1} resolution)
(g) After 5 min annealing at 15 K (1 cm^{-1} resolution)
(h) After 5 min annealing at 20 K (1 cm^{-1} resolution)
(i) After 5 min annealing at 25 K (1 cm^{-1} resolution)
(j) After 10 min broadband photolysis (1 cm^{-1} resolution)
(l) After 5 min annealing to 30 K (1 cm^{-1} resolution)

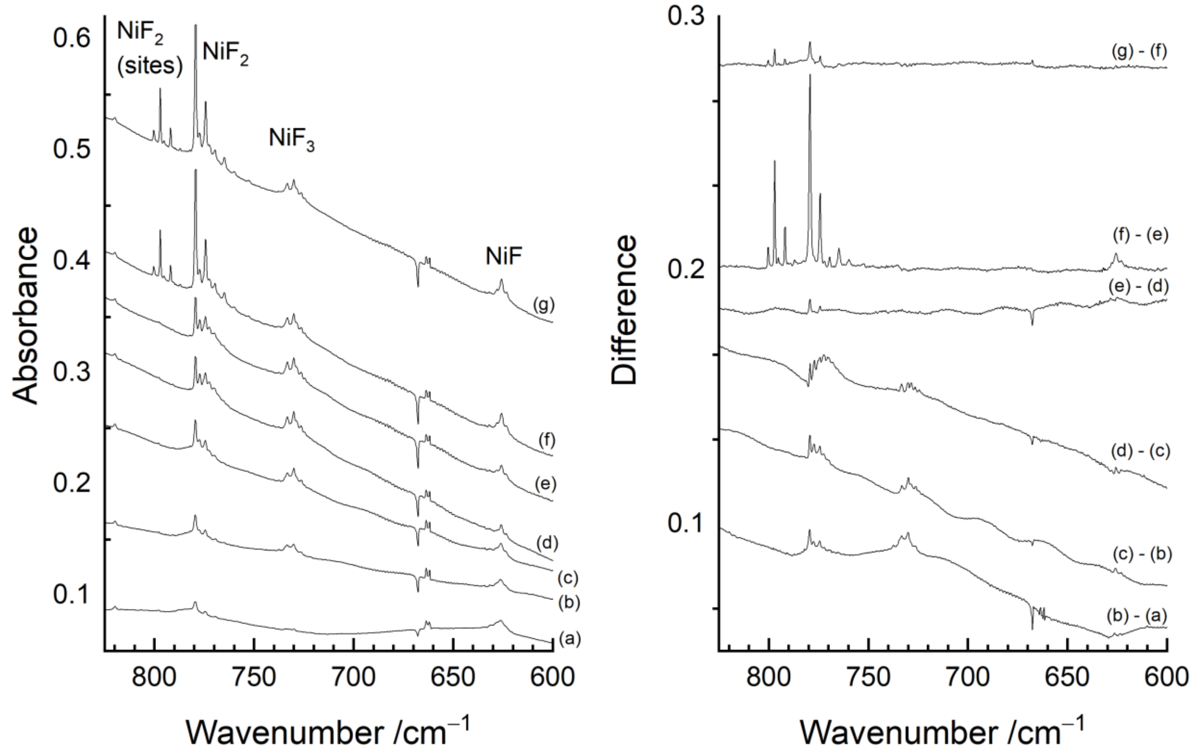

Figure S3. IR spectra (left) and difference spectra (right) of the reaction products of thermally evaporated Ni atoms in 0.5% F_2/ Ar matrix.

(a) After deposition (1 cm^{-1} resolution)
(b) After 5 min annealing at 15 K (1 cm^{-1} resolution)
(c) After 5 min annealing at 20 K (1 cm^{-1} resolution)
(d) After 5 min annealing at 25 K (1 cm^{-1} resolution)
(e) After 10 min visible photolysis (1 cm^{-1} resolution)
(f) After 10 min 410-200 nm photolysis (1 cm^{-1} resolution)
(g) After 10 min broadband photolysis (1 cm^{-1} resolution)

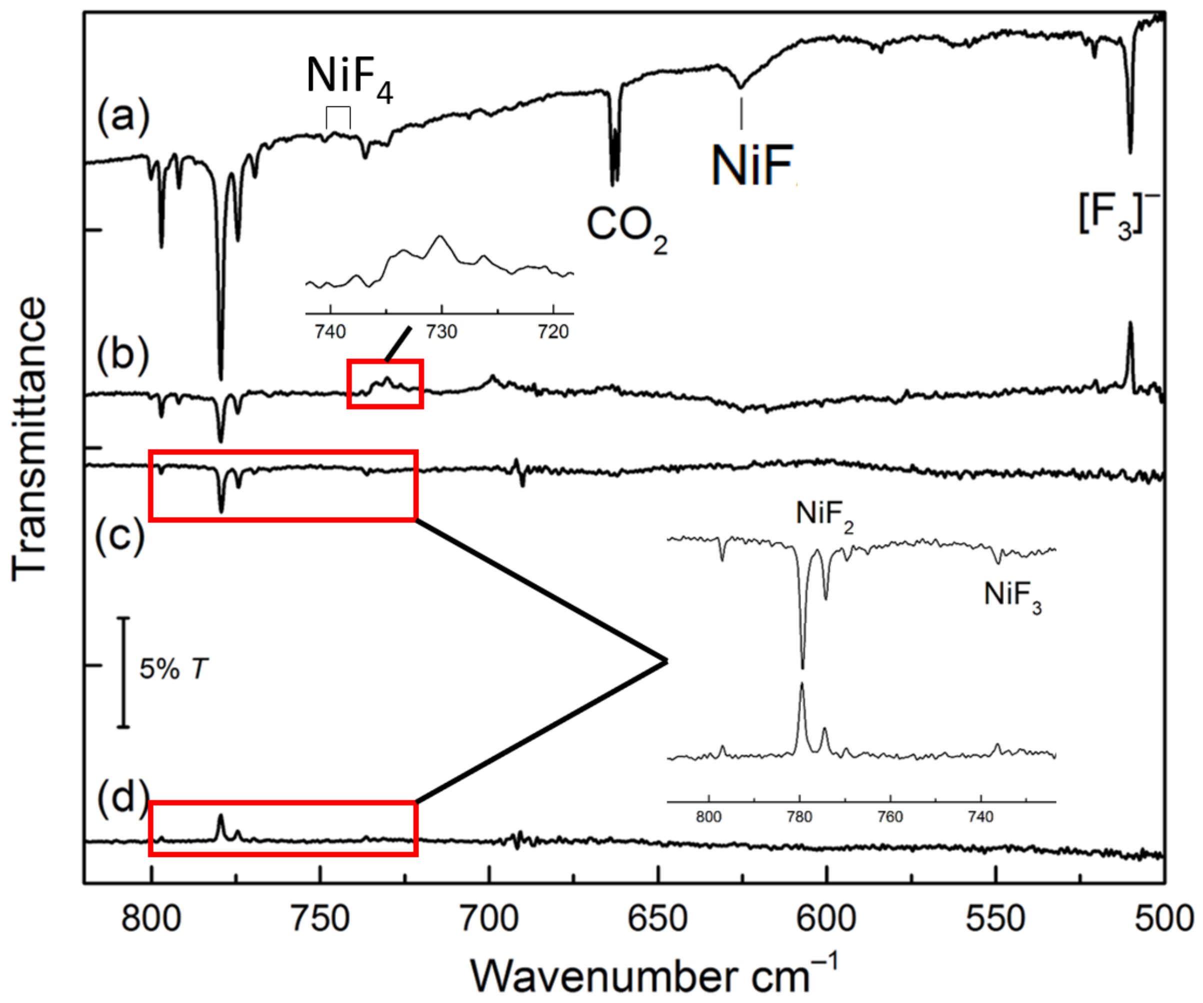

Figure S4. IR spectrum of the reaction products obtained from laser-ablated Ni atoms with 0.5% F_2 seeded in excess argon: (a) co-deposited for 180 min at 12 K, (b) difference spectra after λ = 273 nm (LED) radiation for 40 min, (c) λ = 193 nm laser radiation for 2 min, and subsequent (d) λ = 193 nm laser radiation for 3 min.

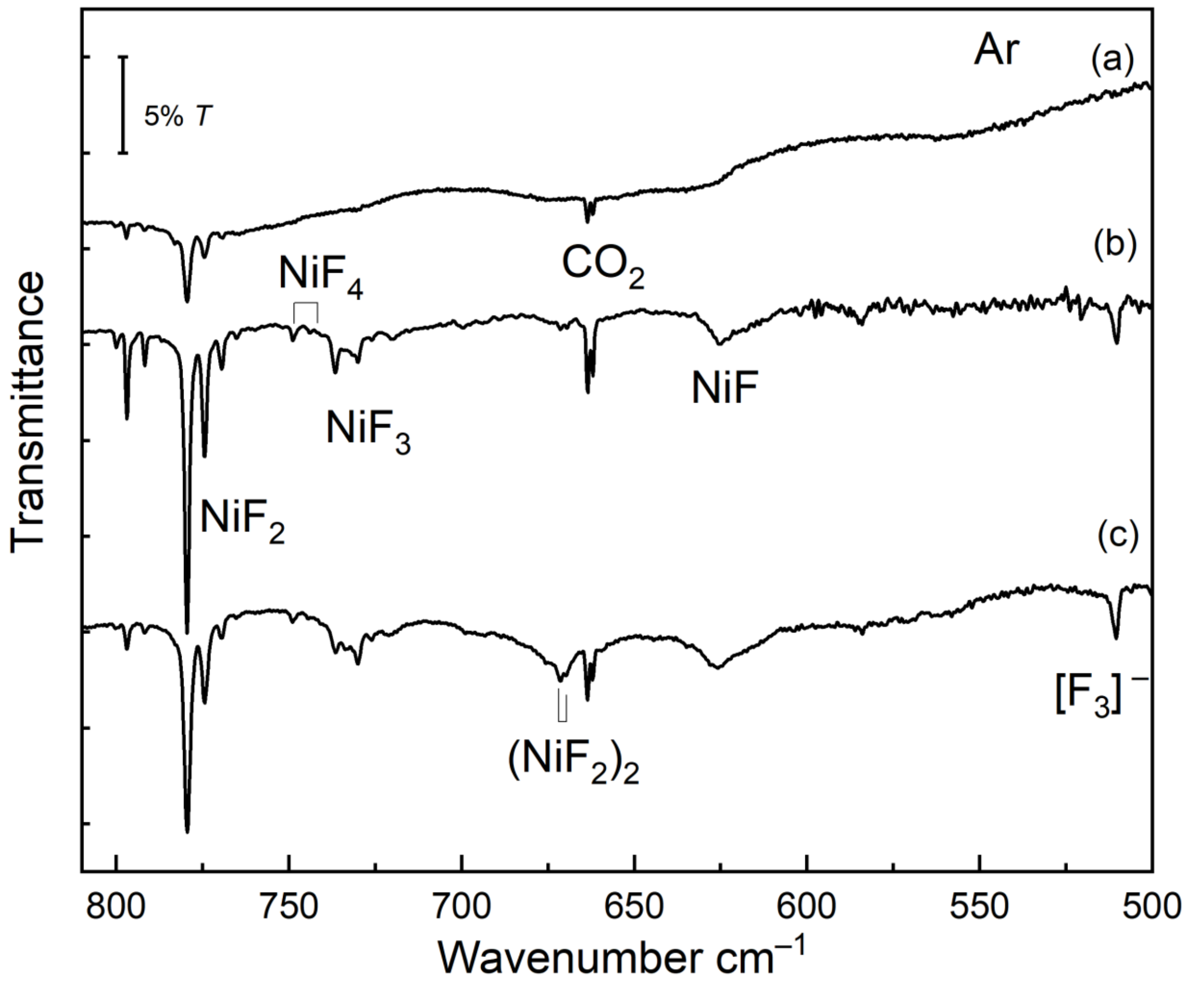

Figure S5. IR spectra of the reaction products obtained from laser-ablated Ni atoms with different concentration of F_2 seeded in excess argon: (a) Ni + 0.1% F_2 co-deposited at 5 K for 80 min, (b) Ni + 0.5% F_2 co-deposited at 15 K for 60 min and (c) Ni + 1% F_2 co-deposited at 5 K for 40 min.

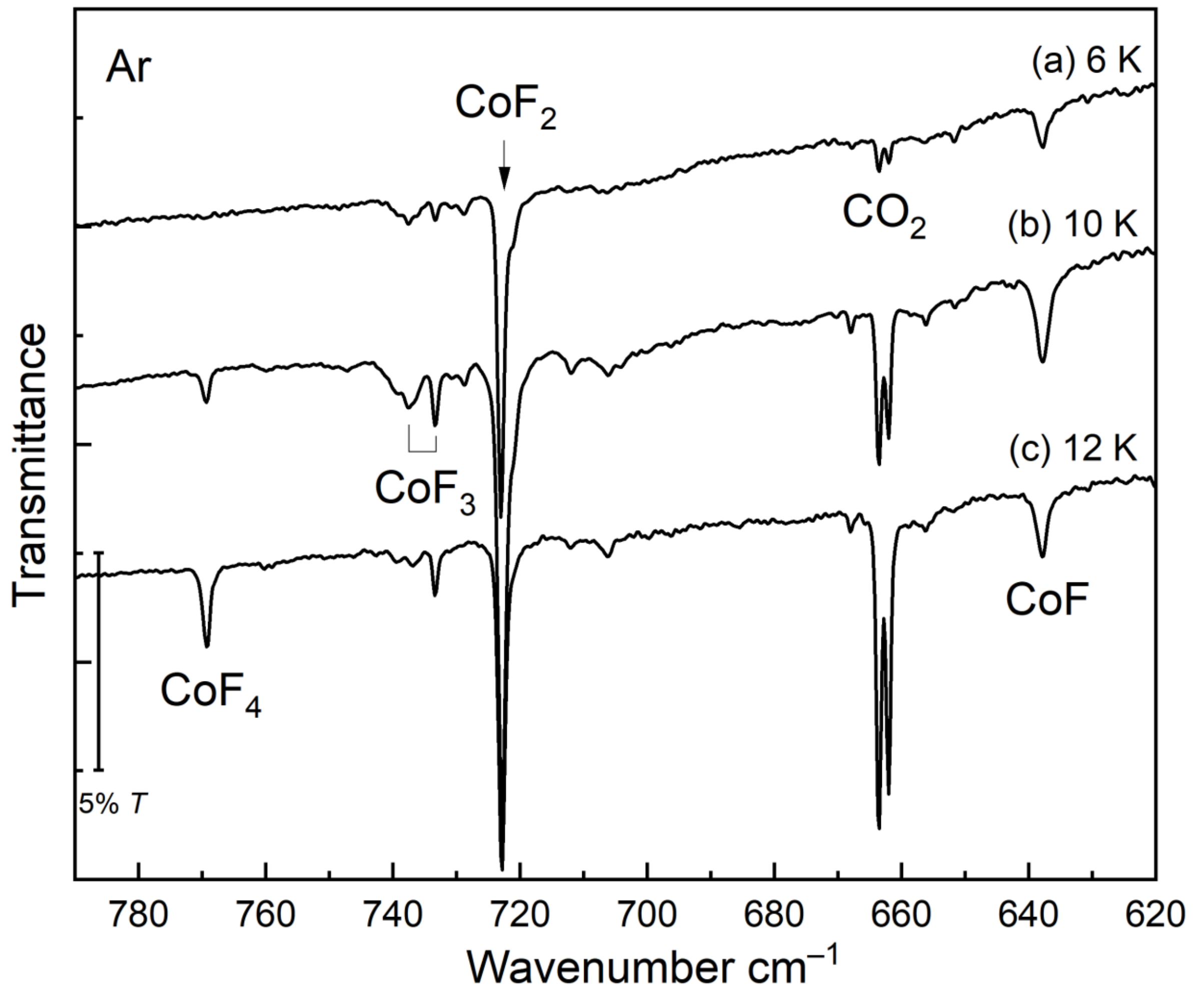

Figure S6. IR Spectra of the reaction products of laser-ablated Co with F_2 seeded in excess argon co-deposited for 60 min: (a) 0.5% F_2 in Ar deposited at 6 K, (b) 1% F_2 in Ar deposited at 10 K, and (c) 0.5% F_2 in Ar deposited at 12 K.

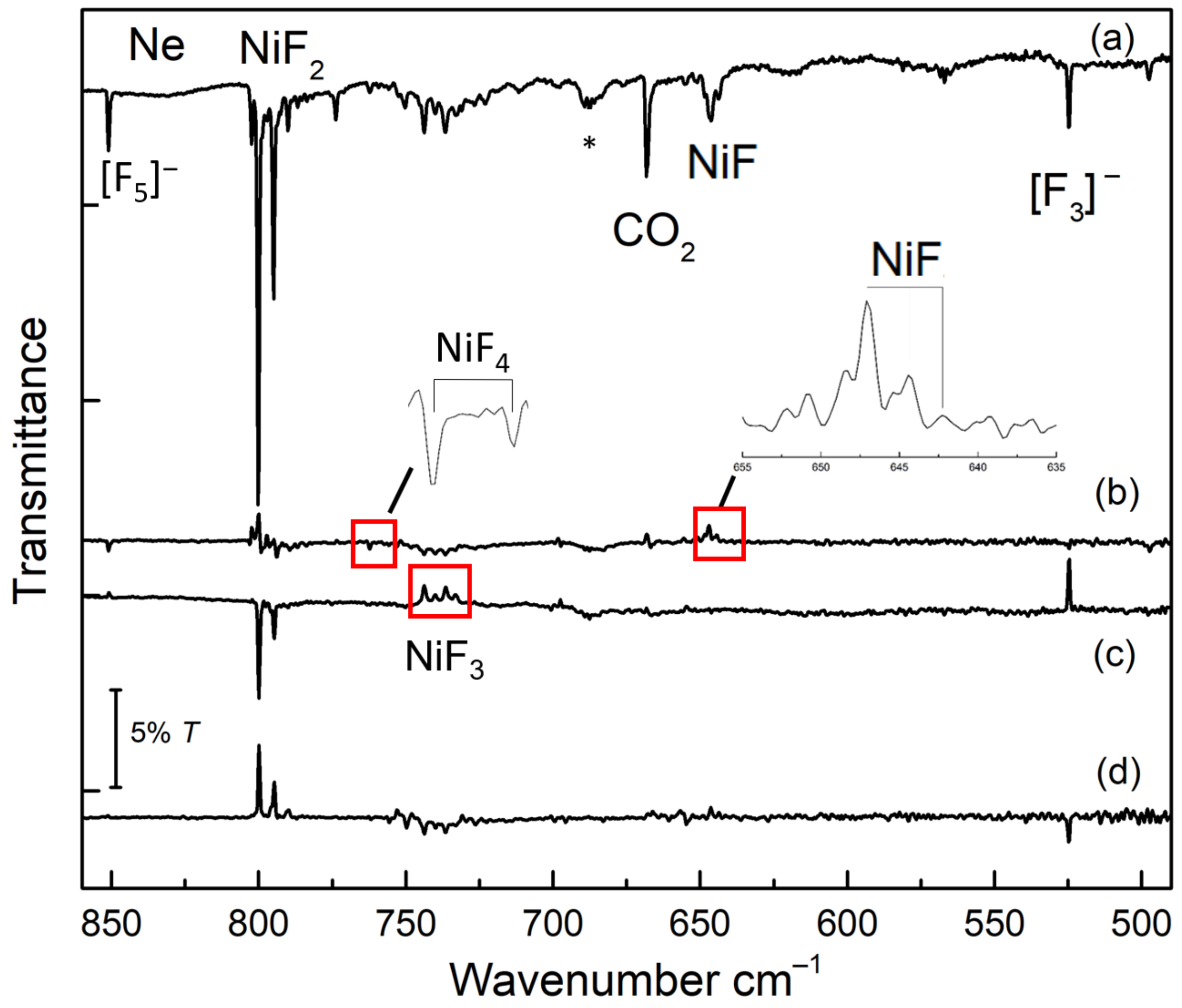

Figure S7. IR spectra of the reaction products of laser-ablated Ni atoms with 0.1% F_2 seeded in excess neon: (a) co-deposited for 120 min at 5 K, (b) difference spectra after annealing to 8 K, followed by (c) λ = 266 nm laser radiation for 15 min, and (d) subsequent annealing to 11 K.

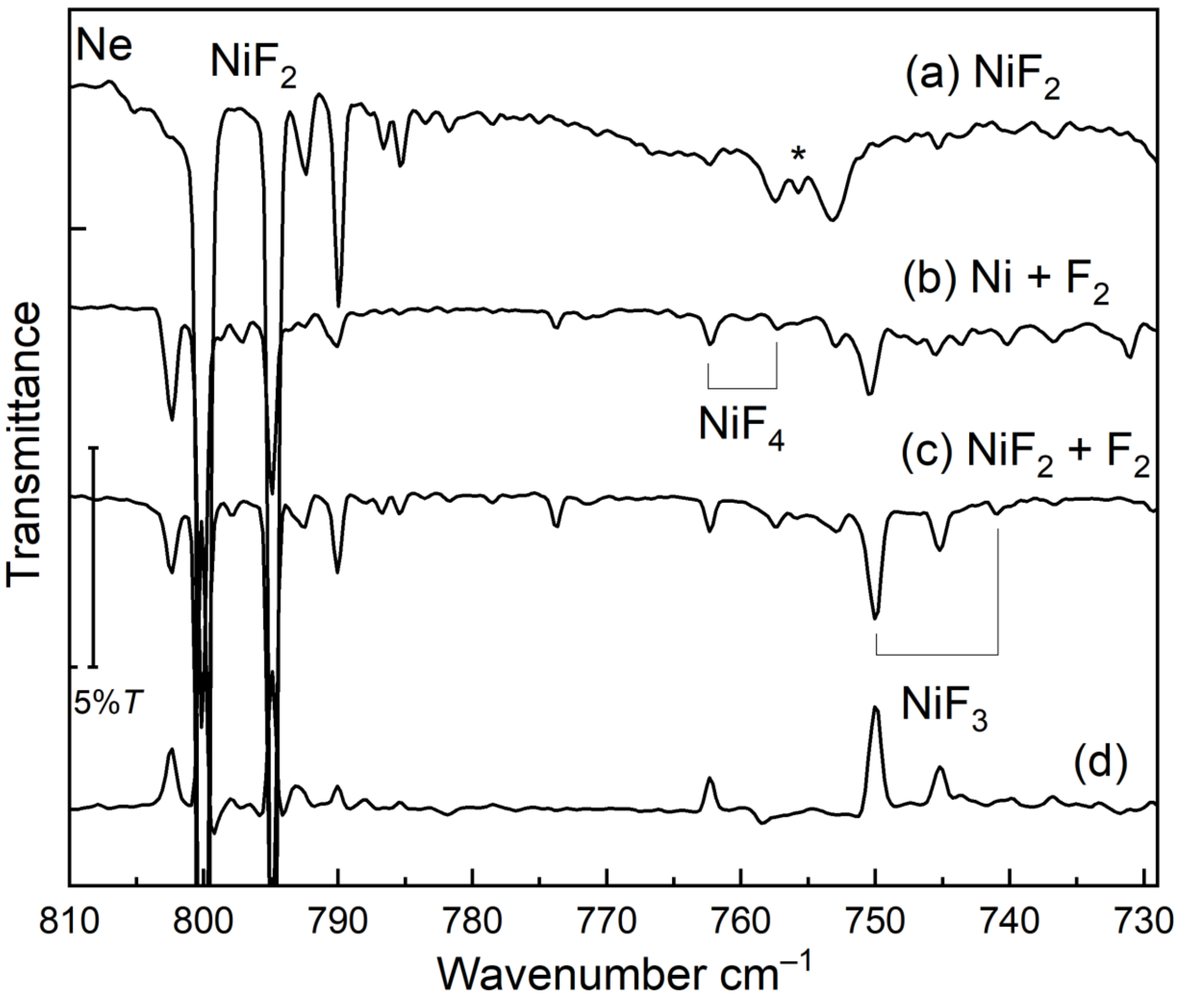

Figure S8. IR spectra of the reaction products obtained from laser-ablated (a) NiF_2, (b) Ni + 0.05% F_2 and (c) NiF_2 + 0.05% F_2 seeded in excess neon. And (d) difference spectrum obtained after λ = 193 nm (laser) radiation for 11 min. An unassigned feature is marked by an asterisk.

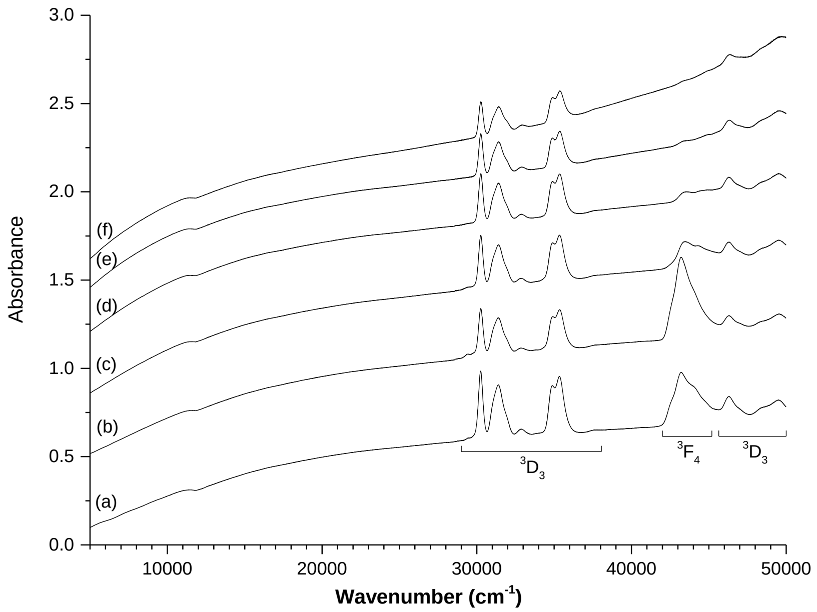

Figure S9. Electronic absorption spectra of Ni atoms in an Ar matrix, (a) on deposition, (b) after broadband photolysis, (c) after annealing to 15 K, (d) after annealing to 20 K, (e) after annealing to 25 K, (f) after annealing to 30 K.

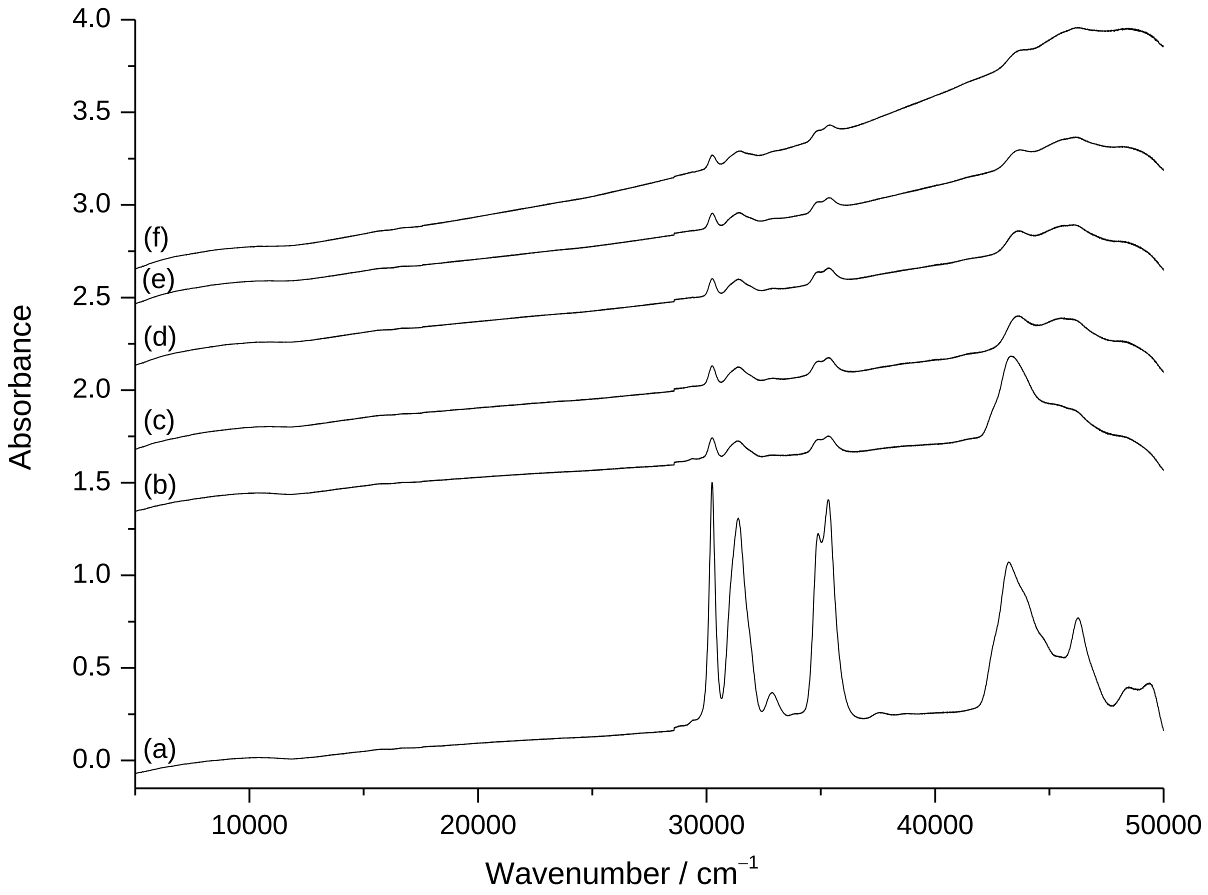

Figure S10. Electronic absorption spectra of Ni atoms in 1%F_2/Ar matrix, (a) on deposition, (b) after broadband photolysis, (c) after annealing to 15 K, (d) after annealing to 20 K, (e) after annealing to 25 K, (f) after annealing to 30 K.

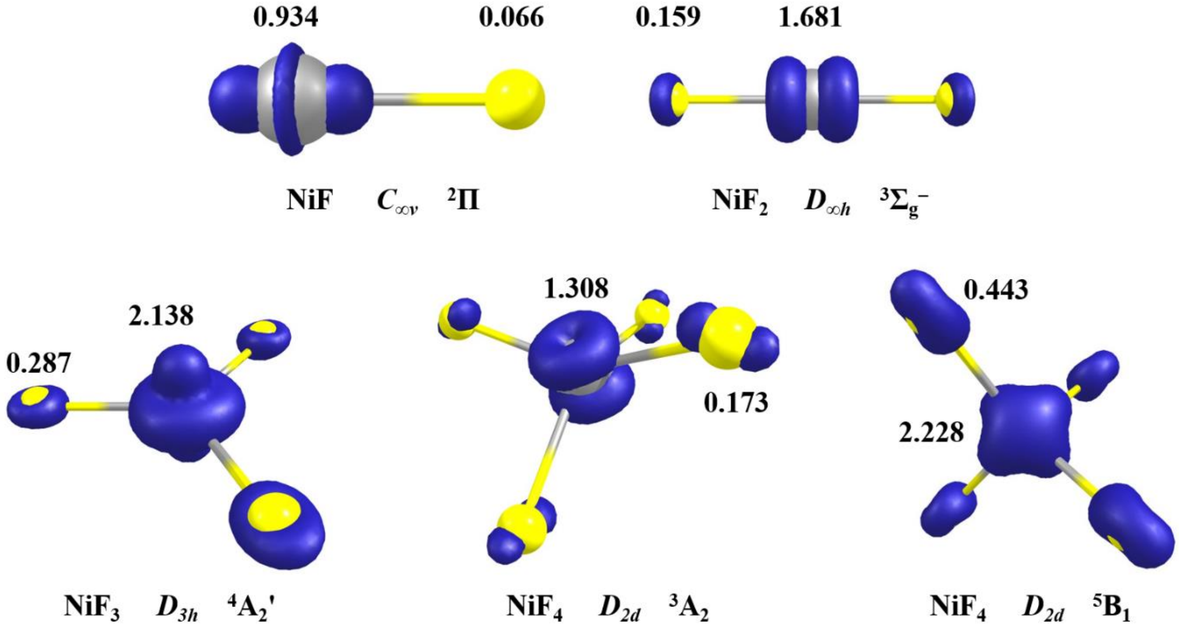

Figure S11. Spin densities of NiF_n (n = 1-4) computed at the B3LYP/AVTZ level of theory (iso-surface = 0.08 electron a.u.$^{-3}$).

Part 3. Supporting Tables

Table S3.1. Experimental vibrational frequencies of nickel fluorides (in cm^{-1}) observed on thermal evaporation of nickel in the presence of elemental fluorine.

	Obs.	SVFF Calculated. (assuming 180° bond angle)	Literature Values [1a]	Literature Values [1b]
$^{58}NiF_2$	779.4	779.4	779.7	780.0
$^{60}NiF_2$	774.3	774.2	774.7	775.0
$^{61}NiF_2$		771.8		
$^{62}NiF_2$	769.2	769.4	770.0	770.3
$^{64}NiF_2$	(764.9†)	764.8	765.5	

† Although this band position is close to the expected value, the band intensity is too high. This feature is therefore more likely to be a matrix site.

$^{58}NiF_2$	797.0	797.0		Extra site 1
$^{60}NiF_2$	791.9	791.7		
$^{61}NiF_2$		789.2		
$^{62}NiF_2$		786.8		
$^{64}NiF_2$		782.1		
$^{58}NiF_2$	800.3	800.3		Extra site 2
$^{60}NiF_2$	795.1	795.0		
$^{61}NiF_2$		792.5		
$^{62}NiF_2$		790.0		
$^{64}NiF_2$		785.3		

Ref. [1]

Table S3.2. Electronic absorptions (cm^{-1}) and assignments for Ni atoms isolated in Ar matrix.

This work	Assignment Ref [2]	This work	Assignment Ref [2]
30250 cm^{-1}	z $^3P_2 \leftarrow {}^3D_3$	42640 cm^{-1} (sh)	x $^3D_3 \leftarrow {}^3F_4$
31050 cm^{-1} (sh)	z $^5F_4 \leftarrow {}^3D_3$	43210 cm^{-1}	y $^3G_5 \leftarrow {}^3F_4$
31380 cm^{-1}	z $^3F_4 \leftarrow {}^3D_3$	44050 cm^{-1} (sh)	x $^3F_3 \leftarrow {}^3F_4$
32870 cm^{-1}	z $^3G_5 \leftarrow {}^3F_4$	46300 cm^{-1}	x $^3P_2 \leftarrow {}^3D_3$
34880 cm^{-1}	y $^3F_4 \leftarrow {}^3D_3$	48470 cm^{-1}	
35350 cm^{-1}	y $^3D_3 \leftarrow {}^3D_3$	49500 cm^{-1}	
37580 cm^{-1} (vw)	y $^1F_3 \leftarrow {}^3D_3$		

Ref. [2]

Table S3.3. Experimental vibrational frequencies of nickel fluorides (in cm^{-1}) observed by laser ablation of nickel in the presence of elemental fluorine.

Species	Ground state (Sym.)	Exp.[a]			
		Ne (Relative Int.)	Assignm.	Ar (Relative Int.)	Assignm.
NiF_2	$^3\Sigma_g^-$($D_{\infty h}$)	802.4 (w)	Matrix site	800.2 (w)	Matrix site
		800.1 (vs)	^{58}Ni, Σ_u^+	797.0 (s)	Matrix site
		797.1 (w)	Matrix site	791.9 (w)	Matrix site
		794.9 (vs)	^{60}Ni, Σ_u^+	779.5 (vs)	^{58}Ni, Σ_u^+
		792.5 (vw)	Matrix site	774.4 (vs)	^{60}Ni, Σ_u^+
		790.1 (s)	^{62}Ni, Σ_u^+	769.3 (s)	^{62}Ni, Σ_u^+
		786.7 (vw)	Matrix site	765.4 (vw)	Matrix site
		785.5 (vw)	Matrix site		
		783.5 (vw)	Matrix site		
		781.8 (vw)	Matrix site		
NiF_3	$^4A_2'$(D_{3h})	750.2 (w)	Matrix site	736.5 (s)	^{58}Ni, E′
		746.7 (vw)	Matrix site	733.9 (vw)	Matrix site
		745.2 (w)	Matrix site	732.9 (vw)	Matrix site
		743.8 (s)	^{58}Ni, E′	731.7 (w)	^{60}Ni, E′
		740.0 (s)	^{60}Ni, E′	730.2 (w)	Matrix site
		736.6 (s)	Matrix site		
		732.9 (s)	Matrix site		
		731.0 (w)	Matrix site		
NiF_4	$^3A_2$(D_{2d})	762.3 (w)	^{58}Ni, E	749.1 (s)	^{58}Ni, E
		760.6 (vw)	Matrix site	746.9 (vw)	Matrix site
		759.2 (vw)	Matrix site	744.0 (vw)	^{60}Ni, E
		757.2 (w)	^{60}Ni, E	743.2 (vw)	Matrix site
		755.8 (vw)	Matrix site		

[a] Relative band intensity (in parentheses) are described as very strong (vs), strong (s), weak (w) and very weak (vw), respectively.

Table S3.4. Electronic states, structural parameters (Å, deg) and electronic energy differences (kJ mol^{-1}) of selected states of NiF_3 and NiF_4.

	Electronic state (Sym.)	Bond lengths [Å] [a] Ni–F/Ni–F′	Angle [°] [a] F–Ni–F	$\Delta E^0_{CCSD(T)}$[a]	$\Delta E^0_{CCSD(T)}$[b]	ΔE^0_{CASPT2}[c]
NiF_3	$^2A'$ (C_{2v})	1.717/1.721	163.0	53.1	58.5	--
	$^4A_2'$ (D_{3h})	1.722	120.0	0.0	0.0	--
NiF_4	$^1A_1$ (D_{2d})	1.709/1.700	159.8	15.0	31.8	--
	$^3A_2$ (D_{2d})	1.700/1.700	137.6	0.0	0.0	22.7
	$^5A_1$ (D_{2d})	1.709/1.709	114.8	17.7	25.4	0.0
	$^5B_1$ (D_{2d})	1.715/1.715	105.6	15.4	24.6	2.1

[a] CCSD(T)/AVTZ, [b] CCSD(T)/AVTZ-DK, [c] CAS(14,22)PT2/AVTZ-DK.

Table S3.5. Computed M−F bond length (r(M−F) in Å) and comparison of computed and experimental vibrational frequencies (ν(M−F) in cm^{-1}) of late **TM** fluorides MF_n (M = Fe, Co, Ni and Cu; n = 2-4).

M =	Method/ basis set	MF_2 r(M−F)	Sym./State	MF_3 r(M−F)	Sym./State	MF_4 r(M−F)	Sym./State
Fe[1]	CCSD(T)/AVTZ	1.771	$D_{\infty h}$, $^5\Delta_g$	1.758	D_{3h}, $^6A_1'$	1.715	D_{2d}, $^5B_2$
Co[2]	CCSD(T)/AVTZ	1.741	$D_{\infty h}$, $^4\Delta_g$	1.722	D_{3h}, $^5A_1'$	1.718	T_d, $^6A_1$
Ni[3]	CCSD(T)/AVTZ	1.718	$D_{\infty h}$, $^3\Sigma_g^-$	1.722	D_{3h}, $^4A_2'$	1.700	D_{2d}, $^3A_2$
						1.716	D_{2d}, $^5B_1$
Cu[4]	CCSD(T)/AVTZ	1.716	$D_{\infty h}$, $^2\Sigma^+_g$	1.675/ 1.666	C_{2v}, $^1A_1$	—	—

ν(M−F)	MF_2 calc.	MF_2, exp: Ne (Ar)	MF_3 calc.	MF_3, exp: Ne (Ar)	MF_4 calc.	MF_4, exp: Ne (Ar)
Fe[1]	594.8 (Σ^+_g)	—	663.6 (A_1')	—	660.0 (B_2)	651.9 (—)
	751.8 (Σ^+_u)	752.5 (730.5)	751.2 (E′)	743.6 (728.5)	791.4 (E)	778.6 (757)
Co[2]	598.3 (Σ^+_g)	—	673.7 (A_1')	—	676.0 (A_1)	—
	788.9 (Σ^+_u)	745.8 (723.5)	754.4 (E′)	748.2 (737.2)	751.4 (T_2)	— (767.8)
Ni[3]	613.3 (Σ^+_g)	—	667.7 (A_1')	—	645.0 (B_2)	—
	805.3 (Σ^+_u)	800.1 (779.5)	766.7 (E′)	743.8 (736.5)	767.2 (E)	762.3 (749.1)
Cu[4]			582.1 (A_1)	—		
	636.1 (Σ^+_g)	—	649.7 (A_1)	—		—
	776.2 (Σ^+_u)	766.0 (743.1)	801.1 (B_2)	786.0 (762.3)		

[1]: Ref. [3]; [2]: Ref. [1b, 4]; [3]: This work; [4]: Ref. [5].

The molecular trifluorides of M = Fe, Co, and Ni all adopt D_{3h} structures, and their stretching fundamentals were found in Ne matrices in a very narrow range between 743.6 cm^{-1} (Fe),[3] 748.2 cm^{-1} (Co),[6] and 743.8 cm^{-1} (Ni, this work). For a series of molecules with similar structures one expects a strong correlation between stretching frequencies and bond lengths,[7] a trend that also applies to CoF_3 with similar computed bond length at the CCSD(T) level. Due to a strong Jahn Teller-induced spin crossover,[5, 8] this series cannot be extended to the $3d^8$ electron configuration of CuF_3 with its singlet T-type (C_{2v}) molecular structure.

3.6. References to Part 3

[1] a) D. E. Milligan, M. E. Jacox, J. D. McKinley, *J. Chem. Phys.* **1965**, *42*, 902-905; b) J. W. Hastie, R. H. Hauge, J. L. Margrave, *High Temp. Sci.* **1969**, *1*, 76-85.
[2] M. Vala, M. Eyring, J. Pyka, J. C. Rivoal, C. Grisolia, *J. Chem. Phys.* **1985**, *83*, 969-974.
[3] T. Schlöder, T. Vent-Schmidt, S. Riedel, *Angew. Chem. Int. Ed.* **2012**, *51*, 12063–12067.
[4] a) V. N. Bukhmarina, A. Y. Gerasimov, Y. B. Predtechenskii, *Vib. Spectrosc*. **1992**, *4*, 91–94; b) J. V. Rau, S. Nunziante Cesaro, N. S. Chilingarov, G. Balducci, *Inorg. Chem.* **1999**, *38*, 5695–5697.
[5] X. Wang, L. Andrews, F. Brosi, S. Riedel, *Chem. Eur. J.* **2013**, *19*, 1397–1409.
[6] V. N. Bukhmarina, A. Y. Gerasimov, Y. B. Predtechenskii, *Vib. Spectrosc.* **1992**, *4*, 91-94.
[7] M. Hargittai, *Chem. Rev.* **2000**, *100*, 2233-2301.
[8] M. Hargittai, *Acc. Chem. Res.* **2009**, *42*, 453-462.

Part 4. CASSCF, MRCI, MRCI+Q and CCSD(T) calculations on NiF ($^2\Pi$)

Table S4.1. Energy (E_h), equilibrium distance (r_{eq}), dipole moment (μ) and harmonic vibrational wavenumber (ω_e) for the $^2\Pi$ state of NiF computed at different non-relativistic (NREL) levels of theory.

$^2\Pi$						
	**CASSCF(9,6)**					
	Energy (E_h)		r_{eq} (Å)	μ (D)	ω_e (cm^{-1}) ^{58}Ni	ω_e (cm^{-1}) ^{60}Ni
**VDZ**	-1606.326749	6	1.7822	3.1833	659.24	656.52
**VTZ**	-1606.361065	6	1.7758	3.1281	659.54	656.82
**VQZ**	-1606.373154	6	1.7774	3.1359	657.58	654.87
**AVDZ**	-1606.338679	6	1.7871	3.2023	647.94	645.28
**AVTZ**	-1606.365463	6	1.7798	3.1483	653.24	650.55
**AVQZ**	-1606.374112	6	1.7796	3.1405	652.88	650.20
	**MRCI**					
	Energy (E_h)		r_{eq} (Å)	μ (D)	ω_e (cm^{-1}) ^{58}Ni	ω_e (cm^{-1}) ^{60}Ni
**VDZ**	-1606.746762	6	1.7377	2.6952	668.87	666.11
**VTZ**	-1606.887415	6	1.7340	2.7549	668.62	665.86
**VQZ**	-1606.945252	6	1.7342	2.7937	668.19	665.44
**AVDZ**	-1606.784939	6	1.7502	2.8463	652.98	650.29
**AVTZ**	-1606.907369	6	1.7387	2.8066	659.59	656.87
**AVQZ**	-1606.953286	6	1.7364	2.7993	662.48	659.76
	**MRCI+Q**					
	Energy (E_h)		r_{eq} (Å)	μ (D)	ω_e (cm^{-1}) ^{58}Ni	ω_e (cm^{-1}) ^{60}Ni
**VDZ**	-1606.783186	6	1.7265	-	668.59	665.84
**VTZ**	-1606.932510	6	1.7233	-	668.40	665.65
**VQZ**	-1606.995155	6	1.7239	-	667.10	664.36
**AVDZ**	-1606.826167	6	1.7412	-	650.11	647.43
**AVTZ**	-1606.955843	6	1.7287	-	657.17	654.46
**AVQZ**	-1607.0046010	6	1.7263	-	660.44	657.72
	**RCCSD(T)**					
	Energy (E_h)		r_{eq} (Å)	μ (D)	ω_e (cm^{-1}) ^{58}Ni	ω_e (cm^{-1}) ^{60}Ni
**VDZ**	-1606.818070	2	1.7522	-	631.04	628.44
**VTZ**	-1606.979444	2	1.7476	-	625.82	623.24
**VQZ**	-1607.049289	2	1.7517	-	620.71	618.15
**AVDZ**	-1606.867681	2	1.7713	-	599.87	597.40
**AVTZ**	-1607.008795	2	1.7573	-	610.01	607.49
**AVQZ**	-1607.061389	2	1.7556	-	612.98	610.46

Table S4.2. Energy (E_h), equilibrium distance (r_{eq}), dipole moment (μ) and harmonic vibrational wavenumber (ω_e) for the $^2\Pi$ state of NiF computed at different levels of theory using the second-order Douglas-Kroll-Hess (DK) Hamiltonian.

	$^2\Pi$					
	**CASSCF(9,6)**					
	Energy (E_h)		r_{eq} (Å)	μ (D)	ω_e (cm^{-1}) ^{58}Ni	ω_e (cm^{-1}) ^{60}Ni
**VDZ-DK**	-1618.608329	6	1.7768	3.1865	660.35	657.63
**VTZ-DK**	-1618.643020	6	1.7696	3.1232	662.13	659.40
**VQZ-DK**	-1618.655582	6	1.7714	3.1356	659.57	656.85
**AVDZ-DK**	-1618.620069	6	1.7817	3.2052	649.32	646.65
**AVTZ-DK**	-1618.647226	6	1.7738	3.1492	655.18	652.48
**AVQZ-DK**	-1618.656488	6	1.7735	3.1416	654.91	652.22
	**MRCI**					
	Energy (E_h)		r_{eq} (Å)	μ (D)	ω_e (cm^{-1}) ^{58}Ni	ω_e (cm^{-1}) ^{60}Ni
**VDZ-DK**	-1619.030885	7	1.7329	2.6936	670.08	667.32
**VTZ-DK**	-1619.171487	5	1.7279	2.7450	671.76	668.99
**VQZ-DK**	-1619.229889	5	1.7283	2.7881	670.50	667.74
**AVDZ-DK**	-1619.068266	7	1.7454	2.8421	654.03	651.34
**AVTZ-DK**	-1619.190860	5	1.7328	2.8008	661.95	659.22
**AVQZ-DK**	-1619.237773	6	1.7304	2.7948	665.03	662.29
	**MRCI+Q**					
	Energy (E_h)		r_{eq} (Å)	μ (D)	ω_e (cm^{-1}) ^{58}Ni	ω_e (cm^{-1}) ^{60}Ni
**VDZ-DK**	-1619.067879	7	1.7220	-	669.35	666.59
**VTZ-DK**	-1619.217352	5	1.7175	-	671.32	668.55
**VQZ-DK**	-1619.280554	6	1.7183	-	669.15	666.39
**AVDZ-DK**	-1619.110131	6	1.7368	-	650.73	648.05
**AVTZ-DK**	-1619.240038	6	1.7230	-	659.35	656.63
**AVQZ-DK**	-1619.289829	6	1.7206	-	662.79	660.06
	**RCCSD(T)**					
	Energy (E_h)		r_{eq} (Å)	μ (D)	ω_e (cm^{-1}) ^{58}Ni	ω_e (cm^{-1}) ^{60}Ni
**VDZ-DK**	-1619.096523	1	1.7303	-	654.08	651.38
**VTZ-DK**	-1619.258333	1	1.7243	-	654.52	651.83
**VQZ-DK**	-1619.328346	1	1.7285	-	646.96	644.30
**AVDZ-DK**	-1619.144877	1	1.7482	-	623.50	620.93
**AVTZ-DK**	-1619.286528	1	1.7334	-	636.56	633.94
**AVQZ-DK**	-1619.340073	1	1.7320	-	639.13	636.50

Part 5. RHF, MP2, CISD and CCSD(T) calculations on NiF_2 ($^3\Sigma_g^-$, $^1\Sigma_g^+$)

Table S5.1. Energy (E_h), bond length (r_{eq}), and relative term energy (T_e) for the $^3\Sigma_g^-$ and $^1\Sigma_g^+$ terms of NiF_2 computed at different non-relativistic (NREL) levels of theory.

R(O)HF	$^3\Sigma_g^-$		$^1\Sigma_g^+$		
	r_{eq} (Å)	Energy (E_h)	r_{eq} (Å)	Energy (E_h)	T_e (eV)
**VDZ**	1.7611	-1705.837755	1.7332	-1705.771689	1.80
**VTZ**	1.7536	-1705.904095	1.7279	-1705.839311	1.76
**VQZ**	1.7550	-1705.926137	1.7295	-1705.861711	1.75
**AVDZ**	1.7633	-1705.860322	1.7360	-1705.794606	1.79
**AVTZ**	1.7568	-1705.912072	1.7308	-1705.847250	1.76
**AVQZ**	1.7567	-1705.927729	1.7308	-1705.863190	1.76

MP2	$^3\Sigma_g^-$		$^1\Sigma_g^+$		
	r_{eq} (Å)	Energy (E_h)	r_{eq} (Å)	Energy (E_h)	T_e (eV)
**VDZ**			1.7145	-1706.453080	
**VTZ**			1.7103	-1706.712780	
**VQZ**			1.7099	-1706.817960	
**AVDZ**			1.7237	-1706.527694	
**AVTZ**			1.7142	-1706.749992	
**AVQZ**			1.7117	-1706.833505	

CISD	$^3\Sigma_g^-$		$^1\Sigma_g^+$		
	r_{eq} (Å)	Energy (E_h)	r_{eq} (Å)	Energy (E_h)	T_e (eV)
**VDZ**	1.7322	-1706.432427	1.7189	-1706.375038	1.56
**VTZ**	1.7263	-1706.657366	1.7125	-1706.603239	1.47
**VQZ**	1.7264	-1706.743930	1.7116	-1706.691703	1.42
**AVDZ**	1.7388	-1706.494782	1.7246	-1706.437435	1.56
**AVTZ**	1.7295	-1706.685771	1.7150	-1706.632185	1.46
**AVQZ**	1.7280	-1706.754987	1.7127	-1706.702873	1.42

(R)CCSD(T)	$^3\Sigma_g^-$		$^1\Sigma_g^+$		
	r_{eq} (Å)	Energy (E_h)	r_{eq} (Å)	Energy (E_h)	T_e (eV)
**VDZ**	1.7219	-1706.525796	1.7297	-1706.472318	1.46
**VTZ**	1.7140	-1706.783420	1.7222	-1706.733757	1.35
**VQZ**	1.7140	-1706.884515	1.7211	-1706.837584	1.28
**AVDZ**	1.7300	-1706.603283	1.7369	-1706.551129	1.42
**AVTZ**	1.7180	-1706.821771	1.7258	-1706.773506	1.31
**AVQZ**	1.7160	-1706.899708	1.7230	-1706.853144	1.27

Table S5.2. Energy (E_h), bond length (r_{eq}), and relative term energy (T_e) for the $^3\Sigma_g^-$ and $^1\Sigma_g^+$ terms of NiF_2 computed at different levels of theory using the second-order Douglas-Kroll-Hess (DK) Hamiltonian.

**R(O)HF**	$^3\Sigma_g^-$		$^1\Sigma_g^+$		
	r_{eq} (Å)	Energy (E_h)	r_{eq} (Å)	Energy (E_h)	T_e (eV)
**VDZ-DK**	1.7512	-1718.206105	1.7300	-1718.137576	1.86
**VTZ-DK**	1.7428	-1718.273440	1.7244	-1718.205997	1.84
**VQZ-DK**	1.7443	-1718.296040	1.7261	-1718.228944	1.83
**AVDZ-DK**	1.7534	-1718.228349	1.7329	-1718.160157	1.86
**AVTZ-DK**	1.7461	-1718.281014	1.7273	-1718.213577	1.84
**AVQZ-DK**	1.7443	-1718.296040	1.7273	-1718.230366	1.79
**MP2**	$^3\Sigma_g^-$		$^1\Sigma_g^+$		
	r_{eq} (Å)	Energy (E_h)	r_{eq} (Å)	Energy (E_h)	T_e (eV)
**VDZ-DK**			1.7122	-1718.821154	
**VTZ-DK**			1.7076	-1719.081265	
**VQZ-DK**			1.7073	-1719.187201	
**AVDZ-DK**			1.7218	-1718.894677	
**AVTZ-DK**			1.7114	-1719.117621	
**AVQZ-DK**			1.7089	-1719.202585	
**CISD**	$^3\Sigma_g^-$		$^1\Sigma_g^+$		
	r_{eq} (Å)	Energy (E_h)	r_{eq} (Å)	Energy (E_h)	T_e (eV)
**VDZ-DK**	1.7215	-1718.803878	1.7162	-1718.742090	1.68
**VTZ-DK**	1.7146	-1719.029167	1.7094	-1718.970664	1.59
**VQZ-DK**	1.7149	-1719.116345	1.7086	-1719.059806	1.54
**AVDZ-DK**	1.7284	-1718.865017	1.7223	-1718.803502	1.67
**AVTZ-DK**	1.7178	-1719.056756	1.7119	-1718.998873	1.58
**AVQZ-DK**	1.7163	-1719.127209	1.7096	-1719.070831	1.53
**(R)CCSD(T)**	$^3\Sigma_g^-$		$^1\Sigma_g^+$		
	r_{eq} (Å)	Energy (E_h)	r_{eq} (Å)	Energy (E_h)	T_e (eV)
**VDZ-DK**	1.7109	-1718.898611	1.7268	-1718.839553	1.61
**VTZ-DK**	1.7021	-1719.157149	1.7189	-1719.101702	1.51
**VQZ-DK**	1.7023	-1719.258811	1.7180	-1719.206120	1.43
**AVDZ-DK**	1.7193	-1718.974904	1.7344	-1718.917411	1.56
**AVTZ-DK**	1.7060	-1719.194486	1.7225	-1719.140492	1.47
**AVQZ-DK**	1.7041	-1719.273763	1.7196	-1719.221480	1.42

Table S5.3. NiF_2 ($^1\Sigma_g^+$) Vibrational Analysis at the non-relativistic (NREL) CISD level of theory.

$^1\Sigma_g^+$								
	**CISD**							
	^{58}Ni				^{60}Ni			
**VDZ**	B_{2u}	B_{3u}	A_g	B_{1u}	B_{2u}	B_{3u}	A_g	B_{1u}
Wavenumbers (cm^{-1})	227.68	227.68	668.81	781.27	226.18	226.18	668.81	776.10
Intensities (km/mol)	0.00	0.00	0.00	72.72	0.00	0.00	0.00	73.69
Intensities [relative]	0.00	0.00	0.00	100.00	0.00	0.00	0.00	100.00
**VTZ**	B_{3u}	B_{2u}	A_g	B_{1u}	B_{3u}	B_{2u}	A_g	B_{1u}
Wavenumbers (cm^{-1})	233.66	233.66	679.63	788.71	232.12	232.12	679.63	783.49
Intensities (km/mol)	0.00	0.00	0.00	80.58	0.00	0.00	0.00	81.66
Intensities [relative]	0.00	0.00	0.00	100.00	0.00	0.00	0.00	100.00
**VQZ**	B_{2u}	B_{3u}	A_g	B_{1u}	B_{2u}	B_{3u}	A_g	B_{1u}
Wavenumbers (cm^{-1})	233.79	233.79	680.55	791.40	232.25	232.25	680.54	786.16
Intensities (km/mol)	0.00	0.00	0.00	81.75	0.00	0.00	0.00	82.84
Intensities [relative]	0.00	0.00	0.00	100.00	0.00	0.00	0.00	100.00
**AVDZ**	B_{2u}	B_{3u}	A_g	B_{1u}	B_{2u}	B_{3u}	A_g	B_{1u}
Wavenumbers (cm^{-1})	225.83	225.83	663.98	770.91	224.30	224.30	663.96	765.80
Intensities (km/mol)	0.00	0.00	0.00	83.46	0.00	0.00	0.00	84.58
Intensities [relative]	0.00	0.00	0.00	100.00	0.00	0.00	0.00	100.00
**AVTZ**	B_{3u}	B_{2u}	A_g	B_{1u}	B_{3u}	B_{2u}	A_g	B_{1u}
Wavenumbers (cm^{-1})	231.67	231.67	674.89	784.34	230.13	230.13	674.90	779.16
Intensities (km/mol)	0.00	0.00	0.00	82.93	0.00	0.00	0.00	84.04
Intensities [relative]	0.00	0.00	0.00	100.00	0.00	0.00	0.00	100.00
**AVQZ**	B_{3u}	B_{2u}	A_g	B_{1u}	B_{3u}	B_{2u}	A_g	B_{1u}
Wavenumbers (cm^{-1})	228.74	228.74	675.20	786.77	227.23	227.23	675.20	781.57
Intensities (km/mol)	0.00	0.00	0.00	82.50	0.00	0.00	0.00	83.61
Intensities [relative]	0.00	0.00	0.00	100.00	0.00	0.00	0.00	100.00

Table S5.4. NiF_2 ($^1\Sigma_g^+$) Vibrational Analysis at the CISD level using the second-order Douglas-Kroll-Hess (DK) Hamiltonian.

$^1\Sigma_g^+$								
	CISD							
	^{58}Ni				^{60}Ni			
**VDZ-DK**	B_{2u}	B_{3u}	A_g	B_{1u}	B_{2u}	B_{3u}	A_g	B_{1u}
Wavenumbers (cm^{-1})	226.78	226.79	671.26	785.35	225.28	225.29	671.26	780.15
Intensities (km/mol)	0.00	0.00	0.00	70.83	0.00	0.00	0.00	71.78
Intensities [relative]	0.00	0.00	0.00	100.00	0.00	0.00	0.00	100.00
**VTZ-DK**	B_{2u}	B_{3u}	A_g	B_{1u}	B_{2u}	B_{3u}	A_g	B_{1u}
Wavenumbers (cm^{-1})	232.07	232.07	682.93	794.11	230.54	230.54	682.93	788.85
Intensities (km/mol)	0.00	0.00	0.00	78.91	0.00	0.00	0.00	79.96
Intensities [relative]	0.00	0.00	0.00	100.00	0.00	0.00	0.00	100.00
**VQZ-DK**	B_{2u}	B_{3u}	A_g	B_{1u}	B_{2u}	B_{3u}	A_g	B_{1u}
Wavenumbers (cm^{-1})	231.44	231.45	682.89	796.09	229.91	229.92	682.89	790.82
Intensities (km/mol)	0.00	0.00	0.00	80.01	0.00	0.00	0.00	81.07
Intensities [relative]	0.00	0.00	0.00	100.00	0.00	0.00	0.00	100.00
**AVDZ-DK**	B_{2u}	B_{3u}	A_g	B_{1u}	B_{2u}	B_{3u}	A_g	B_{1u}
Wavenumbers (cm^{-1})	223.35	223.36	665.95	773.70	221.87	221.88	665.95	768.58
Intensities (km/mol)	0.00	0.00	0.00	81.33	0.00	0.00	0.00	82.42
Intensities [relative]	0.00	0.00	0.00	100.00	0.00	0.00	0.00	100.00
**AVTZ-DK**	B_{2u}	B_{3u}	A_g	B_{1u}	B_{2u}	B_{3u}	A_g	B_{1u}
Wavenumbers (cm^{-1})	230.97	230.97	678.30	789.37	229.44	229.44	678.30	784.15
Intensities (km/mol)	0.00	0.00	0.00	81.15	0.00	0.00	0.00	82.23
Intensities [relative]	0.00	0.00	0.00	100.00	0.00	0.00	0.00	100.00
**AVQZ-DK**	B_{3u}	B_{2u}	A_g	B_{1u}	B_{3u}	B_{2u}	A_g	B_{1u}
Wavenumbers (cm^{-1})	225.52	225.53	677.30	791.33	224.03	224.04	677.30	786.09
Intensities (km/mol)	0.00	0.00	0.00	80.80	0.00	0.00	0.00	81.88
Intensities [relative]	0.00	0.00	0.00	100.00	0.00	0.00	0.00	100.00

Table S5.5. NiF_2 ($^1\Sigma_g^+$) Vibrational Analysis at the RCCSD(T) level of theory.

$^1\Sigma_g^+$								
	**CCSD(T)**							
	^{58}Ni				^{60}Ni			
**VDZ**	B_{2u}	B_{3u}	A_g	B_{1u}	B_{2u}	B_{3u}	A_g	B_{1u}
Wavenumbers (cm^{-1})	209.50	209.50	642.29	763.20	208.11	208.11	642.29	758.15
**VTZ**	B_{2u}	B_{3u}	A_g	B_{1u}	B_{3u}	B_{2u}	A_g	B_{1u}
Wavenumbers (cm^{-1})	214.17	214.17	652.57	766.06	212.75	212.75	652.58	760.99
**VQZ**	B_{3u}	B_{2u}	A_g	B_{1u}	B_{3u}	B_{2u}	A_g	B_{1u}
Wavenumbers (cm^{-1})	217.55	217.55	654.55	768.00	216.10	216.10	654.54	762.92
**AVDZ**	B_{3u}	B_{2u}	A_g	B_{1u}	B_{2u}	B_{3u}	A_g	B_{1u}
Wavenumbers (cm^{-1})	207.45	207.45	634.89	746.71	206.07	206.07	634.89	741.77
**AVTZ**	B_{3u}	B_{2u}	A_g	B_{1u}	B_{3u}	B_{2u}	A_g	B_{1u}
Wavenumbers (cm^{-1})	213.59	213.59	646.05	757.86	212.19	212.19	646.06	752.85
**AVQZ**	B_{2u}	B_{3u}	A_g	B_{1u}	B_{2u}	B_{3u}	A_g	B_{1u}
Wavenumbers (cm^{-1})	214.24	214.24	649.67	761.44	212.82	212.82	649.67	756.40
**VDZ-DK**	B_{2u}	B_{3u}	A_g	B_{1u}	B_{2u}	B_{3u}	A_g	B_{1u}
Wavenumbers (cm^{-1})	207.56	207.56	645.13	766.85	206.19	206.19	645.13	761.78
**VTZ-DK**	B_{3u}	B_{2u}	A_g	B_{1u}	B_{3u}	B_{2u}	A_g	B_{1u}
Wavenumbers (cm^{-1})	212.47	212.48	656.83	771.60	211.06	211.07	656.83	766.50
**VQZ-DK**	B_{3u}	B_{2u}	A_g	B_{1u}	B_{3u}	B_{2u}	A_g	B_{1u}
Wavenumbers (cm^{-1})	215.44	215.45	658.12	772.75	214.01	214.02	658.12	767.63
**AVDZ-DK**	B_{3u}	B_{2u}	A_g	B_{1u}	B_{3u}	B_{2u}	A_g	B_{1u}
Wavenumbers (cm^{-1})	204.44	204.44	637.28	749.36	203.09	203.09	637.28	744.40
**AVTZ-DK**	B_{3u}	B_{2u}	A_g	B_{1u}	B_{3u}	B_{2u}	A_g	B_{1u}
Wavenumbers (cm^{-1})	211.37	211.37	649.53	762.62	209.97	209.97	649.53	757.58
**AVQZ-DK**	B_{3u}	B_{2u}	A_g	B_{1u}	B_{2u}	B_{3u}	A_g	B_{1u}
Wavenumbers (cm^{-1})	212.66	212.67	653.49	766.95	211.17	211.17	653.41	761.84

Table S5.6. NiF_2 ($^3\Sigma_g^-$) Vibrational Analysis at the non-relativistic (NREL) CISD level of theory.

$^3\Sigma_g^-$								
	CISD							
	^{58}Ni				^{60}Ni			
**VDZ**	B_{3u}	B_{2u}	A_g	B_{1u}	B_{3u}	B_{2u}	A_g	B_{1u}
Wavenumbers (cm^{-1})	122.29	122.29	617.47	803.16	122.29	122.29	617.47	803.16
Intensities (km/mol)	0.00	0.00	0.00	53.88	0.00	0.00	0.00	53.88
Intensities [relative]	0.00	0.00	0.00	100.00	0.00	0.00	0.00	100.00
**VTZ**	B_{2u}	B_{3u}	A_g	B_{1u}	B_{2u}	B_{3u}	A_g	B_{1u}
Wavenumbers (cm^{-1})	124.00	124.00	620.54	811.25	123.18	123.18	620.54	805.88
Intensities (km/mol)	0.00	0.00	0.00	62.03	0.00	0.00	0.00	62.86
Intensities [relative]	0.00	0.00	0.00	100.00	0.00	0.00	0.00	100.00
**VQZ**	B_{3u}	B_{2u}	A_g	B_{1u}	B_{3u}	B_{2u}	A_g	B_{1u}
Wavenumbers (cm^{-1})	124.51	124.51	621.71	812.79	123.69	123.69	621.71	807.42
Intensities (km/mol)	0.00	0.00	0.00	64.46	0.00	0.00	0.00	65.32
Intensities [relative]	0.00	0.00	0.00	100.00	0.00	0.00	0.00	100.00
**AVDZ**	B_{3u}	B_{2u}	A_g	B_{1u}	B_{3u}	B_{2u}	A_g	B_{1u}
Wavenumbers (cm^{-1})	120.45	120.45	609.82	793.78	119.65	119.65	609.82	788.53
Intensities (km/mol)	0.00	0.00	0.00	65.24	0.00	0.00	0.00	66.11
Intensities [relative]	0.00	0.00	0.00	100.00	0.00	0.00	0.00	100.00
**AVTZ**	B_{2u}	B_{3u}	A_g	B_{1u}	B_{2u}	B_{3u}	A_g	B_{1u}
Wavenumbers (cm^{-1})	122.43	122.43	615.65	805.63	121.62	121.62	615.65	800.30
Intensities (km/mol)	0.00	0.00	0.00	65.11	0.00	0.00	0.00	65.98
Intensities [relative]	0.00	0.00	0.00	100.00	0.00	0.00	0.00	100.00
**AVQZ**	B_{2u}	B_{3u}	A_g	B_{1u}	B_{2u}	B_{3u}	A_g	B_{1u}
Wavenumbers (cm^{-1})	113.79	113.79	618.37	807.58	113.04	113.04	618.37	802.24
Intensities (km/mol)	0.00	0.00	0.00	65.05	0.00	0.00	0.00	65.92
Intensities [relative]	0.00	0.00	0.00	100.00	0.00	0.00	0.00	100.00

Table S5.7. NiF_2 ($^3\Sigma_g^-$) Vibrational Analysis at the CISD level using the second-order Douglas-Kroll-Hess (DK) Hamiltonian.

$^3\Sigma_g^-$								
	CISD							
	^{58}Ni				^{60}Ni			
**VDZ-DK**	B_{2u}	B_{3u}	A_g	B_{1u}	B_{2u}	B_{3u}	A_g	B_{1u}
Wavenumbers (cm^{-1})	133.02	133.02	622.65	818.06	132.14	132.14	622.65	812.65
Intensities (km/mol)	0.00	0.00	0.00	53.28	0.00	0.00	0.00	53.99
Intensities [relative]	0.00	0.00	0.00	100.00	0.00	0.00	0.00	100.00
**VTZ-DK**	B_{3u}	B_{2u}	A_g	B_{1u}	B_{3u}	B_{2u}	A_g	B_{1u}
Wavenumbers (cm^{-1})	133.87	133.88	627.90	823.61	132.99	132.99	627.90	818.16
Intensities (km/mol)	0.00	0.00	0.00	62.06	0.00	0.00	0.00	62.89
Intensities [relative]	0.00	0.00	0.00	100.00	0.00	0.00	0.00	100.00
**VQZ-DK**	B_{2u}	B_{3u}	A_g	B_{1u}	B_{2u}	B_{3u}	A_g	B_{1u}
Wavenumbers (cm^{-1})	135.32	135.32	628.99	824.62	134.42	134.43	628.99	819.16
Intensities (km/mol)	0.00	0.00	0.00	64.25	0.00	0.00	0.00	65.11
Intensities [relative]	0.00	0.00	0.00	100.00	0.00	0.00	0.00	100.00
**AVDZ-DK**	B2u	B_{3u}	A_g	B_{1u}	B_{2u}	B_{3u}	A_g	B_{1u}
Wavenumbers (cm^{-1})	128.35	128.35	615.00	802.44	127.50	127.50	615.00	797.13
Intensities (km/mol)	0.00	0.00	0.00	64.74	0.00	0.00	0.00	65.61
Intensities [relative]	0.00	0.00	0.00	100.00	0.00	0.00	0.00	100.00
**AVTZ-DK**	B_{2u}	B_{3u}	A_g	B_{1u}	B_{2u}	B_{3u}	A_g	B_{1u}
Wavenumbers (cm^{-1})	132.39	132.40	622.71	817.49	131.51	131.52	622.71	812.09
Intensities (km/mol)	0.00	0.00	0.00	64.84	0.00	0.00	0.00	65.70
Intensities [relative]	0.00	0.00	0.00	100.00	0.00	0.00	0.00	100.00
**AVQZ-DK**	B_{3u}	B_{2u}	A_g	B_{1u}	B_{3u}	B_{2u}	A_g	B_{1u}
Wavenumbers (cm^{-1})	125.04	125.06	625.35	819.61	124.21	124.23	625.35	814.19
Intensities (km/mol)	0.00	0.00	0.00	64.82	0.00	0.00	0.00	65.69
Intensities [relative]	0.00	0.00	0.00	100.00	0.00	0.00	0.00	100.00

Table S5.8. NiF_2 ($^3\Sigma_g^-$) Vibrational Analysis at the RCCSD(T) level of theory.

$^3\Sigma_g^-$								
	**RCCSD(T)**							
	^{58}Ni				^{60}Ni			
**VDZ**	B_{2u}	B_{3u}	A_g	B_{1u}	B_{2u}	B_{3u}	A_g	B_{1u}
Wavenumbers (cm^{-1})	123.59	123.59	613.67	808.93	122.78	122.78	613.67	803.58
**VTZ**	B_{3u}	B_{2u}	A_g	B_{1u}	B_{3u}	B_{2u}	A_g	B_{1u}
Wavenumbers (cm^{-1})	125.35	125.35	619.28	814.31	124.52	124.52	619.28	808.92
**VQZ**	B_{3u}	B_{2u}	A_g	B_{1u}	B_{3u}	B_{2u}	A_g	B_{1u}
Wavenumbers (cm^{-1})	126.87	126.87	619.63	815.33	126.03	126.03	619.63	809.94
**AVDZ**	B_{2u}	B_{3u}	A_g	B_{1u}	B_{2u}	B_{3u}	A_g	B_{1u}
Wavenumbers (cm^{-1})	123.35	123.35	604.43	791.71	122.54	122.54	604.43	786.47
**AVTZ**	B_{2u}	B_{3u}	A_g	B_{1u}	B_{2u}	B_{3u}	A_g	B_{1u}
Wavenumbers (cm^{-1})	124.73	124.73	613.34	805.31	123.90	123.90	613.34	799.98
**AVQZ**	B_{3u}	B_{2u}	A_g	B_{1u}	B_{3u}	B_{2u}	A_g	B_{1u}
Wavenumbers (cm^{-1})	119.48	119.48	613.23	807.94	118.69	118.69	613.23	802.60
**VDZ-DK**	B_{3u}	B_{2u}	A_g	B_{1u}	B_{3u}	B_{2u}	A_g	B_{1u}
Wavenumbers (cm^{-1})	137.97	137.97	622.39	820.74	137.05	137.06	622.39	815.31
**VTZ-DK**	B_{3u}	B_{2u}	A_g	B_{1u}	B_{3u}	B_{2u}	A_g	B_{1u}
Wavenumbers (cm^{-1})	137.13	137.13	629.35	827.51	136.22	136.22	629.35	822.04
**VQZ-DK**	B_{2u}	B_{3u}	A_g	B_{1u}	B_{2u}	B_{3u}	A_g	B_{1u}
Wavenumbers (cm^{-1})	135.69	135.69	628.14	826.97	134.79	134.80	628.14	821.50
**AVDZ-DK**	B_{2u}	B_{3u}	A_g	B_{1u}	B_{2u}	B_{3u}	A_g	B_{1u}
Wavenumbers (cm^{-1})	132.17	132.17	611.45	800.52	131.29	131.30	611.45	795.23
**AVTZ-DK**	B_{2u}	B_{3u}	A_g	B_{1u}	B_{2u}	B_{3u}	A_g	B_{1u}
Wavenumbers (cm^{-1})	135.65	135.65	621.09	818.13	134.75	134.75	621.09	812.72
**AVQZ-DK**	B_{3u}	B_{2u}	A_g	B_{1u}	B_{3u}	B_{2u}	A_g	B_{1u}
Wavenumbers (cm^{-1})	141.83	141.84	626.81	822.68	140.89	140.90	626.81	817.24

Part 6. RHF, MRCI and CCSD(T) calculations on NiF_3 ($^2A_1$ (C_{2v}) and $^4A_2'$ (D_{3h}))

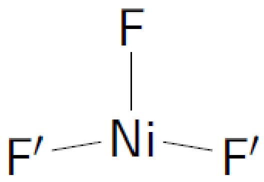

Table S6.1. Energy (E_h), equilibrium distance (Å), and dipole moment (μ) for the $^2A_1$ (C_{2v}) state of NiF_3 computed at different levels of theory.

$^2A_1$					
	NREL/RHF				
	Ni-F (Å)	Ni-F′ (Å)	F-Ni-F′ (°)	Energy (E_h)	μ (D)
VDZ	1.681	1.697	101.274	-1805.144	2.5977
AVDZ	1.682	1.696	101.856	-1805.1791	2.4398
VTZ	1.675	1.691	101.484	-1805.2493	2.5517
AVTZ	1.678	1.693	101.623	-1805.261	2.4719
VQZ	1.677	1.692	101.599	-1805.2818	2.5129
AVQZ	1.678	1.693	101.645	-1805.2843	2.4652
	DK/RHF				
	Ni-F (Å)	Ni-F′ (Å)	F-Ni-F′ (°)	Energy (E_h)	μ (D)
VDZ-DK	1.678	1.694	101.483	-1817.6065	2.5676
AVDZ-DK	1.678	1.694	102.086	-1817.6414	2.4042
VTZ-DK	1.672	1.688	101.691	-1817.7134	2.5178
AVTZ-DK	1.675	1.690	101.834	-1817.7247	2.438
VQZ-DK	1.674	1.689	101.802	-1817.7466	2.4797
AVQZ-DK	1.674	1.690	101.842	-1817.7490	2.4332
	NREL/CISD (MRCI)				
	Ni-F (Å)	Ni-F′ (Å)	F-Ni-F′ (°)	Energy (E_h)	μ (D)
VDZ	1.678	1.697	99.196	-1805.9093	2.4836
AVDZ	1.677	1.697	99.704	-1805.9909	2.3953
VTZ	1.665	1.686	99.385	-1806.215	2.5436
AVTZ	1.667	1.688	99.362	-1806.2505	2.4888
VQZ	1.664	1.685	99.423	-1806.328	2.5371
	DK/CISD (MRCI)				
	Ni-F (Å)	Ni-F′ (Å)	F-Ni-F′ (°)	Energy (E_h)	μ (D)
VDZ-DK	1.673	1.693	99.466	-1818.371	2.4511
AVDZ-DK	1.672	1.693	100.028	-1818.452	2.3517
VTZ-DK	1.661	1.682	99.647	-1818.6782	2.5059
AVTZ-DK	1.663	1.684	99.642	-1818.713	2.4492

Continued on next page

	NREL/RCCSD(T)				
	Ni-F (Å)	Ni-F′ (Å)	F-Ni-F′ (°)	Energy (E_h)	μ (D)
VDZ	1.740	1.732	98.874	-1806.0796	---
AVDZ	1.733	1.732	99.044	-1806.1843	---
VTZ	1.715	1.718	98.751	-1806.4355	---
AVTZ	1.717	1.721	98.520	-1806.4854	---
	DK/RCCSD(T)				
	Ni-F (Å)	Ni-F′ (Å)	F-Ni-F′ (°)	Energy (E_h)	μ (D)
VDZ-DK	1.730	1.725	99.131	-1818.5396	---
AVDZ-DK	1.724	1.726	99.384	-1818.6435	---
VTZ-DK	1.707	1.712	99.014	-1818.8971	---
AVTZ-DK	1.708	1.715	98.819	-1818.946	---

Table S6.2. NiF_3 ($^2A_1$, C_{2v}) vibrational analysis at the CISD and the RCCSD(T) levels of theory.

$^2A_1$							
	CISD (MRCI)						
AVTZ	B_2	B_1	A_1	A_1	A_1	B_2	
Wavenumber (cm^{-1})	231.45(13)	248.64(0)	255.80(28)	708.72(19)	800.75(94)	840.47(92)	^{58}Ni
	231.17(12)	246.74(0)	254.84(28)	708.46(19)	797.42(92)	834.68(93)	^{60}Ni
AVTZ-DK	B_2	B_1	A_1	A_1	A_1	B_2	
Wavenumber (cm^{-1})	232.78(12)	249.61(0)	256.15(28)	715.12(16)	808.57(93)	844.87(90)	^{58}Ni
	231.50(11)	247.70(0)	255.19(28)	714.87(17)	805.17(90)	839.07(91)	^{60}Ni
	NREL/RCCSD(T)						
AVTZ	B_2	B_1	A_1	A_1	A_1	B_2	
Wavenumber (cm^{-1})	151.74	212.26	231.31	624.62	683.20	781.81	^{58}Ni
	151.44	210.64	230.37	624.42	680.41	776.64	^{60}Ni
	DK/RCCSD(T)						
AVTZ-DK	B_2	B_1	A_1	A_1	A_1	B_2	
Wavenumber (cm^{-1})	163.53	217.84	238.35	638.15	698.20	789.14	^{58}Ni
	163.14	216.16	237.38	638.01	695.28	783.93	^{60}Ni

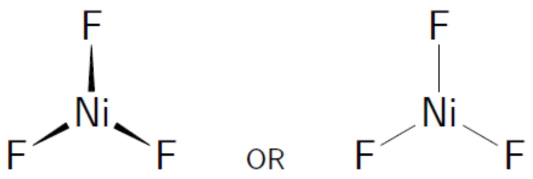

Table S6.3. Energy (E_h), equilibrium distance (Å), and dipole moment (μ) for the $^4A_2'$ (D_{3h}) state of NiF_3 computed at different levels of theory.

$^4A_2'$				
	NREL/RHF			
	Ni-F (Å)	F-Ni-X (°)	Energy (E_h)	μ (D)
VDZ	1.714	88.557	-1805.2111	0.3934
AVDZ	1.714	88.414	-1805.2504	0.4174
VTZ	1.708	89.360	-1805.3204	0.1702
AVTZ	1.711	90.919	-1805.3334	0.2404
VQZ	1.709	90.587	-1805.3538	0.1548
AVQZ	1.710	89.208	-1805.3566	0.2067
	DK/RHF			
	Ni-F (Å)	F-Ni-X (°)	Energy (E_h)	μ (D)
VDZ-DK	1.711	88.457	-1817.6746	0.4182
AVDZ-DK	1.711	88.349	-1817.7136	0.4315
VTZ-DK	1.705	89.263	-1817.7854	0.1943
AVTZ-DK	1.708	90.973	-1817.7979	0.2525
VQZ-DK	1.706	89.293	-1817.8193	0.1848
AVQZ-DK	1.708	89.136	-1817.8220	0.2237
	NREL/CISD (MRCI)			
	Ni-F (Å)	F-Ni-X (°)	Energy (E_h)	μ (D)
VDZ	1.705	90.000	-1805.9495	0.0000
AVDZ	1.706	90.000	-1806.0367	0.0000
VTZ	1.697	90.000	-1806.2594	0.0000
AVTZ	1.699	90.000	-1806.2959	0.0000
	DK/CISD (MRCI)			
	Ni-F (Å)	F-Ni-X (°)	Energy (E_h)	μ (D)
VDZ-DK	1.701	90.000	-1818.4130	0.0000
AVDZ-DK	1.703	90.000	-1818.4995	0.0000
VTZ-DK	1.693	90.000	-1818.7243	0.0000
AVTZ-DK	1.696	90.000	-1818.7600	0.0000

Continued on next page

	NREL/RCCSD(T)			
	Ni-F (Å)	F-Ni-X (°)	Energy (E_h)	μ (D)
VDZ	1.729	87.619	-1806.0982	---
AVDZ	1.730	87.218	-1806.2065	---
VTZ	1.719	87.317	-1806.4559	---
AVTZ	1.720	90.000	-1806.50874	---
	DK/RCCSD(T)			
	Ni-F (Å)	F-Ni-X (°)	Energy (E_h)	μ (D)
VDZ-DK	1.724	87.580	-1818.5601	---
AVDZ-DK	1.726	87.214	-1818.6679	---
VTZ-DK	1.713	87.310	-1818.9195	---
AVTZ-DK	1.715	90.000	-1818.9714	---

Table S6.4. NiF_3 ($^4A_2'$, D_{3h}) vibrational analysis at the CISD and the RCCSD(T) levels of theory.

$^4A_2'$							
	CISD (MRCI)						
AVTZ	E′	E′	A_2''	A_1'	E′	E′	
Wavenumber (cm^{-1})	195.12(23)	195.10(3)	298.44(0)	726.16(0)	814.75(193)	814.71(64)	^{58}Ni
	194.60(23)	194.58(3)	295.97(0)	726.16(0)	810.13(190)	810.08(65)	^{60}Ni
AVTZ-DK	E′	E′	A_2''	A_1'	E′	E′	
Wavenumber (cm^{-1})	195.84(23)	195.78(3)	298.37(0)	728.49(0)	817.15(190)	817.13(63)	^{58}Ni
	195.34(23)	195.26(3)	295.91(0)	728.50(0)	812.53(186)	812.51(64)	^{60}Ni
	NREL/RCCSD(T)						
VTZ	E′	E′	A_2''	A_1'	E′	E′	
Wavenumber (cm^{-1})	182.85	184.68	208.24	668.87	770.84	770.83	^{58}Ni
	183.47	183.75	206.61	669.23	766.75	766.61	^{60}Ni
	NREL/RCCSD(T)						
AVTZ	E′	E′	A_2''	A_1'	E′	E′	
Wavenumber (cm^{-1})	170.32	173.65	355.02	661.37	762.60	762.95	^{58}Ni
	169.85	173.16	352.10	661.37	758.32	758.67	^{60}Ni
	DK/RCCSD(T)						
AVTZ-DK	E′	E′	A_2''	A_1'	E′	E′	
Wavenumber (cm^{-1})	173.49	173.67	355.31	670.05	766.67	767.83	^{58}Ni
	173.00	173.18	352.39	670.05	762.38	763.54	^{60}Ni

Part 7. NiF_4

Structural details of NiF_4 (D_{2d})

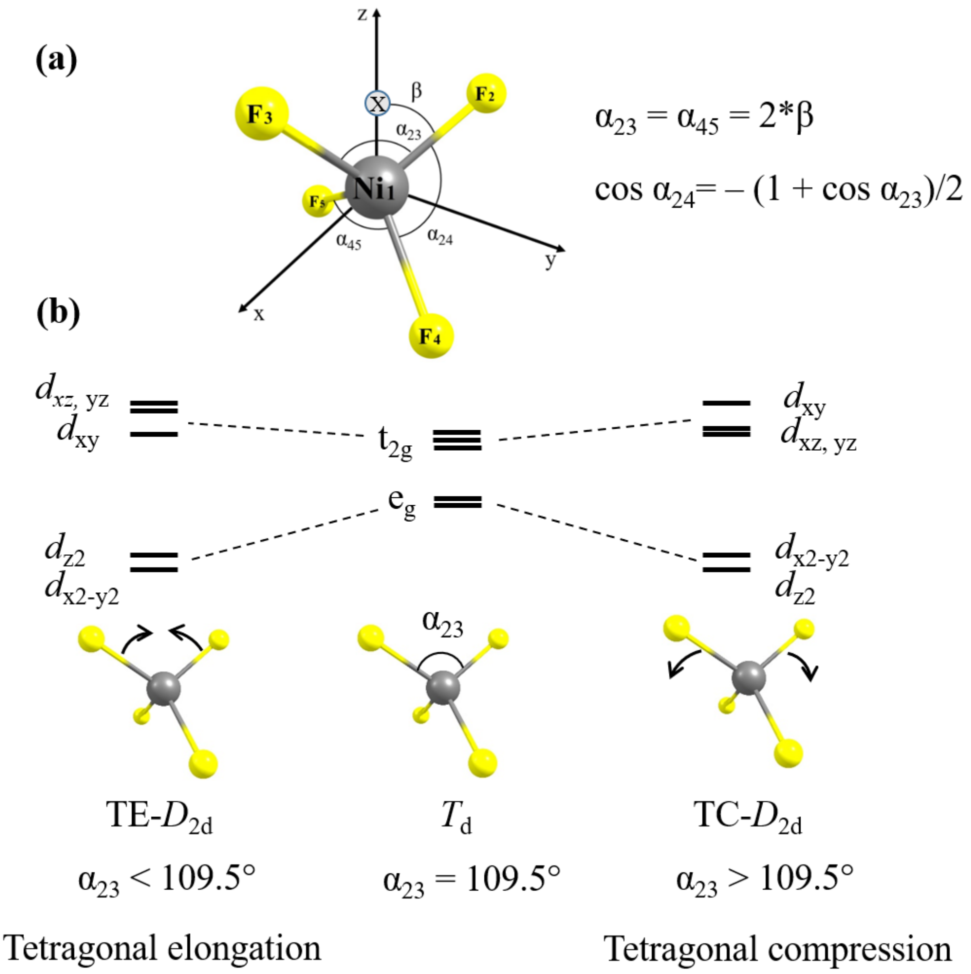

Figure S7.1. (a) The relationship between the valence angles in an D_{2d}-distortet NiF_4 tetrahedron. (b) Evolution of the *d* orbital energy levels for a NiF_4 tetrahedra that are elongated (left, TE-D_{2d}) and compressed (right, TC-D_{2d}) along the *z* direction from the ideal $\alpha_{23} = 109.5°$.

DFT calculation on NiF_4 ($^3A_2$ and $^5B_1$)

Table S7.1. Energy (E_h), equilibrium structure (bond length in Å, angles in deg.) for a tetragonal compressed NiF_4 in the $^3A_2$ (TC-D_{2d}) and $^5B_1$ (TE-D_{2d}) states computed at the DFT level.

	B3LYP/AVTZ			
Point group	State	Ni-F (Å)	X-Ni-F (°)	Energy (E_h)
D_{2d}	$^3A_2$	1.7102	70.53	-1907.8819
D_{2d}	$^5B_1$	1.7373	52.94	-1907.8831

Table S7.2. NiF_4 ($^3A_2$, TC-D_{2d}) and ($^5B_1$, TE-D_{2d}) vibrational analysis at the DFT level.

$^3A_2$										
	B3LYP									
AVTZ	A_1	B_2	E	E	B_1	B_2	A_1	E	E	
Wavenumber (cm^{-1})	104.78(0)	204.73(30)	228.81(6)	228.81(6)	263.83(0)	622.37(23)	638.17(0)	744.29(100)	744.29(100)	^{58}Ni
	104.78(0)	203.14(30)	228.07(6)	228.07(6)	263.83(0)	621.27(22)	638.17(0)	739.61(98)	739.61(98)	^{60}Ni

$^5B_1$										
	B3LYP									
AVTZ	B_1	E	E	A_1	B_2	A_1	E	E	B_2	
Wavenumber (cm^{-1})	67.98(0)	168.21(16)	168.21(16)	182.89(0)	218.26(5)	616.72(0)	659.80(65)	659.80(65)	706.17(40)	^{58}Ni
	67.98(0)	167.37(16)	167.37(16)	182.89(0)	217.39(5)	616.72(0)	656.82(63)	656.82(63)	702.25(39)	^{60}Ni

CCSD(T) and CASPT2 calculations on NiF_4 ($^3A_2$, $^5A_1$ and $^5B_1$)

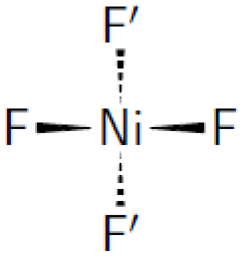

Results for NiF_4 ^3A (C_1)
========================
Open-shell system, 64 electrons
Electron configuration:
- closed shells: a 1-31
- open shells: a 32-33

Table S7.3. Energy (E_h), equilibrium structure (bond length in Å, angles in deg.), and dipole moment (μ) for a tetragonal compressed NiF_4 in the triplet state $^3A_2$ (TC-D_{2d}) computed at different levels of theory.

$^3A_2$						
	DK/CASPT2					
	Ni-F (Å)	Ni-F′ (Å)	X-Ni-F (°)	X-Ni-F′ (°)	Energy (E_h)	μ (D)
VDZ-DK	1.718	1.718	61.8	118.2	-1918.226	0.0019(*)
AVDZ-DK	1.722	1.722	63.0	117.0	-1918.343	0.0005(*)
VTZ-DK	1.707	1.707	63.4	116.6	-1918.665	0.0003(*)
AVTZ-DK	1.718	1.718	76.0	104.0	-1918.736	
	NREL/RCCSD(T)					
	Ni-F (Å)	Ni-F′ (Å)	X-Ni-F (°)	X-Ni-F′ (°)	Energy (E_h)	μ (D)
VDZ	1.721	1.719	64.3	113.9	-1905.647	---
AVDZ	1.713	1.712	68.9	111.0	-1905.779	---
VTZ	1.701	1.700	68.8	111.2	-1906.100	---
AVTZ	1.700	1.700	68.8	111.2	-1906.161	
	DK/RCCSD(T)					
	Ni-F (Å)	Ni-F′ (Å)	X-Ni-F (°)	X-Ni-F′ (°)	Energy (E_h)	μ (D)
VDZ-DK	1.712	1.711	66.8	112.4	-1918.199	---
AVDZ-DK	1.707	1.706	69.6	110.4	-1918.331	---
VTZ-DK	1.694	1.694	69.3	110.6	-1918.655	---
AVTZ-DK	1.700	1.700	68.8	111.2	-1918.719	---

(*) dipole moment at SCF level
F-NI-F = 2 * F-NI-X (F-NI-X < 90 DEG);
F′-NI-F′ = 2 * (180 DEG - F′-NI-X) (F′-NI-X > 90 DEG)

Table S7.4. NiF_4 ($^3A_2$, TC-D_{2d}) vibrational analysis at the RCCSD(T) level.[a]

$^3A_2$										
	NREL/RCCSD(T)									
AVTZ	A_1	A_1	B_2	B_1	A_2	A_1	A_1	B_1	B_2	
Wavenumber (cm^{-1})	115.35	198.06	214.52	216.26	251.80	643.70	644.96	766.93	767.24	^{58}Ni
	115.35	196.63	213.77	215.54	251.80	643.60	643.71	762.24	762.56	^{60}Ni
	DKH/RCCSD(T)									
AVTZ-DK	A_1	A_1	B_2	B_1	A_2	A_1	A_1	B_2	B_1	
Wavenumber (cm^{-1})	111.88	205.94	222.43	227.36	260.38	651.19	654.13	778.50	778.66	^{58}Ni
	111.88	204.38	221.70	226.64	260.38	649.91	654.13	773.71	773.87	^{60}Ni

[a]: Tetragonal compressed tetrahedron. Type and number of normal modes in C_{2v} symmetry.

Table S7.5. Energy (E_h), and equilibrium structure (bond length in Å, angles in deg.) for the quintet states $^5A_1$ (TC-D_{2d}) and $^5B_1$ (TE-D_{2d}) of NiF_4 computed at different levels of theory.

	RCCSD(T)/AVTZ			
Point group	State	Ni-F (Å)	X-Ni-F (°)	Energy (E_h)
D_{2d}	$^5A_1$	1.709	57.4	-1906.1546407
D_{2d}	$^5B_1$	1.715	52.8	-1906.155498
	RCCSD(T)/AVTZ-DK			
Point group	State	Ni-F (Å)	X-Ni-F (°)	Energy (E_h)
D_{2d}	$^5A_1$	1.706	57.1	-1918.7090710
D_{2d}	$^5B_1$	1.710	52.8	-1918.7093652
	CAS(14,22)PT2/AVTZ			
Point group	State	Ni-F (Å)	X-Ni-F (°)	Energy (E_h)
D_{2d}	$^5A_1$	1.740	56.3	-1906.1935213
D_{2d}	$^5B_1$	1.741	53.5	-1906.1927261
	CAS(14,22)PT2/AVTZ-DK			
Point group	State	Ni-F (Å)	X-Ni-F (°)	Energy (E_h)
D_{2d}	$^5A_1$	1.732	56.4	-1918.7448943
D_{2d}	$^5B_1$	1.732	53.4	-1918.7440960

Table S7.6. Vibrational analysis of NiF_4 in the $^5A_1$ ((TC-D_{2d}) and the $^5B_1$ (TE-D_{2d}) electronic states at the RCCSD(T) level.[a]

$^5A_1$ (TC-D_{2d})										
	RCCSD(T)/AVTZ									
	A_2	A_1	A_1	B_1	B_2	A_1	A_1	B_2	B_1	
Wavenumber (cm^{-1})	160.44	164.67	173.51	274.08	275.55	630.16	700.66	793.35	794.14	^{58}Ni
	160.48	163.92	173.57	273.18	274.66	630.17	697.75	788.50	789.30	^{60}Ni
	RCCSD(T)/AVTZ-DK									
	A_1	A_2	A_1	B_1	B_2	A_1	A_1	B_2	B_1	
Wavenumber (cm^{-1})	113.73	121.04	147.06	304.74	311.92	686.63	795.25	858.16	860.03	^{58}Ni
	113.36	121.04	146.98	304.19	310.89	683.71	795.26	852.31	854.00	^{60}Ni

$^5B_1$ (TE-D_{2d})										
	RCCSD(T)/AVTZ									
	B_2	B_1	A_1	A_2	A_1	A_1	B_1	B_2	A_1	
Wavenumber (cm^{-1})	177.14	178.52	188.00	219.35	245.57	666.03	703.62	704.54	752.88	^{58}Ni
	176.22	177.68	188.00	219.35	244.65	666.03	700.37	701.31	748.59	^{60}Ni
	RCCSD(T)/AVTZ-DK									
	B_2	B_1	A_1	A_2	A_1	A_1	B_1	B_2	A_1	
Wavenumber (cm^{-1})	143.99	171.83	173.67	369.73	394.05	670.35	704.46	706.25	939.96	^{58}Ni
	143.04	171.35	173.67	369.73	393.50	670.36	701.18	703.05	932.37	^{60}Ni

[a]: Type and number of normal modes in C_{2v} symmetry.

Table S7.7. NiF_4 ($^3A_2$, TC-D_{2d})

CAS(14,22)PT2/AVTZ-DK

NiF = 1.71758085 ANG
XNiF = 76.03049590 DEGREE

```
CI vector
 =========

 22220 22a 22a 220      0.9163846
 22220 22a 22a 202     -0.1597900
 22220 2a2 22a 2ba     -0.0981272
 22220 22a 2a2 2ba     -0.0981272
 22220 22a 22a 2ba     -0.0780422
 22220 2a2 22a 2ab      0.0668575
 22220 22a 2a2 2ab      0.0668575
 22220 22a 22b 2aa      0.0562241
 22220 22b 22a 2aa      0.0562241
 22220 20a 22a 222     -0.0525154
 22220 22a 20a 222     -0.0525154
 22220 2a2 2a2 220     -0.0510435
 a2220 222 222 2a0     -0.0509495
 22220 a22 22a 2ab      0.0509328
 22220 22a a22 2ab      0.0509328

 TOTAL ENERGIES                        -1917.27316513
```

NATURAL ORBITALS
================

```
  Orbital  Occupation    Energy     Coefficients
    6.1     2.00000    -0.75507     2 2px  -0.64944     2 2py   0.64944
    7.1     1.99272    -0.89060     1 3d2+  0.97882
    8.1     1.99214    -0.70776     2 2pz   0.84279
    9.1     1.98955    -0.79253     1 3d0  -0.71708     2 2px   0.33459     2 2py   0.33459     2 2pz  -0.31139
   10.1     1.97846    -0.83066     1 3d0   0.66500     2 2px   0.41831     2 2py   0.41831     2 2pz  -0.31980
   11.1     0.02812     0.41610     1 1s    1.02181     1 1s    0.34053     1 3d0   0.49641     1 3d0  -0.25919     2 1s   -0.25062
                                    2 2px   0.57957     2 2py   0.57957

    5.2     2.00000    -0.69934     1 2px  -0.26943     2 2px   0.60155     2 2py  -0.74115
    6.2     1.98107    -0.70343     2 2pz   0.89452
    7.2     1.95288    -0.70958     1 2px  -0.44990     1 2px   0.39199     1 3d1-  0.37993     2 2px  -0.68132     2 2py  -0.48742
    8.2     1.04695    -0.44513     1 3d1-  0.88708     2 2px   0.31135     2 2pz  -0.27845

    5.3     2.00000    -0.69934     1 2py  -0.26943     2 2py   0.60155     2 2px  -0.74115
    6.3     1.98107    -0.70343     2 2pz   0.89452
    7.3     1.95288    -0.70958     1 2py  -0.44990     1 2py   0.39199     1 3d1+  0.37993     2 2py  -0.68132     2 2px  -0.48742
    8.3     1.04695    -0.44513     1 3d1+  0.88708     2 2py   0.31135     2 2pz  -0.27845

    5.4     2.00000    -0.66283     2 2py  -0.70676     2 2px   0.70676
    6.4     1.98975    -0.72946     2 2pz   0.82437     2 2py  -0.27310     2 2px  -0.27310
    7.4     1.87341    -0.75984     1 3d2- -0.67521     2 2pz   0.29548     2 2py   0.41881     2 2px   0.41881
    8.4     0.19404    -0.12093     1 3d2- -0.70926     1 3d2-  0.26994     2 2pz  -0.34121     2 2py  -0.50806     2 2px  -0.50806
```

Table S7.8. NiF_4 ($^5A_1$, TC-D_{2d})

CAS(14,22)PT2/AVTZ-DK

NiF = 1.73218347 ANG
XNiF = 56.39831565 DEGREE

```
CI vector
 =========

 222a0 22a 22a 22a      0.9278587
 22a20 22a 22a 2a2      0.1578140
 22a20 2a2 22a 22a      0.1116338
 22a20 22a 2a2 22a      0.1116338
 222a0 2a2 22a 2a2     -0.1060445
 222a0 22a 2a2 2a2     -0.1060445
 222a0 2a2 2a2 22a     -0.1009806
 22a20 2a2 22a 2a2      0.0533694
 22a20 22a 2a2 2a2      0.0533694
 2a220 2a2 22a 22a      0.0513814
 2a220 22a 2a2 22a     -0.0513814

 TOTAL ENERGIES                     -1917.26176050
```

NATURAL ORBITALS
================

```
 Orbital  Occupation    Energy     Coefficients
   6.1     2.00000    -0.71432     2 2px  -0.39709     2 2py  -0.39709     2 2pz   0.75964
   7.1     1.99173    -0.86634     1 3d0   0.66449     2 2px   0.37756     2 2py   0.37756     2 2pz   0.28774
   8.1     1.98567    -0.84685     1 3d0   0.72626     2 2px  -0.27172     2 2py  -0.27172     2 2pz  -0.41113
   9.1     1.93673    -0.76230     1 3d2+  0.48186     2 2px   0.55493     2 2py  -0.55493
  10.1     1.08257    -0.51870     1 3d2+ -0.84807     2 2px   0.37292     2 2py  -0.37292
  11.1     0.02222     0.46448     1 1s    0.93015     1 1s    0.49144     1 1s    0.26098     2 1s   -0.31201     2 2px   0.57430
                                   2 2py   0.57430     2 2pz   0.59008

   5.2     2.00000    -0.68862     2 2px  -0.28194     2 2pz   0.82464     2 2py  -0.40948
   6.2     1.99339    -0.72379     1 2px  -0.46243     1 2px   0.37733     2 2px   0.32887     2 2py  -0.80803
   7.2     1.94411    -0.76893     1 2px  -0.28100     1 3d1-  0.49919     2 2px  -0.70856     2 2pz  -0.32842
   8.2     1.05490    -0.44214     1 3d1-  0.84312     2 2px   0.48092

   5.3     2.00000    -0.68862     2 2py  -0.28194     2 2pz   0.82464     2 2px  -0.40948
   6.3     1.99339    -0.72379     1 2py  -0.46243     1 2py   0.37733     2 2py   0.32887     2 2px  -0.80803
   7.3     1.94411    -0.76893     1 2py  -0.28100     1 3d1+  0.49919     2 2py  -0.70856     2 2pz  -0.32842
   8.3     1.05490    -0.44214     1 3d1+  0.84312     2 2py   0.48092

   5.4     2.00000    -0.71344     1 2pz  -0.32881     1 2pz   0.26730     2 2pz   0.60382     2 2py  -0.49906     2 2px  -0.49906
   6.4     1.99257    -0.65390     2 2py   0.68143     2 2px  -0.68143
   7.4     1.93500    -0.80874     1 2pz  -0.28141     1 3d2-  0.58615     2 2pz  -0.56869     2 2py  -0.32694     2 2px  -0.32694
   8.4     1.06868    -0.46370     1 2pz   0.25941     1 3d2-  0.78521     2 2pz   0.49023     2 2py   0.26285     2 2px   0.26285
```

Table S7.9. NiF_4 ($^5B_1$, TE-D_{2d})

CAS(14,22)PT2/AVTZ-DK

NiF = 1.73243120 ANG
XNiF = 53.44085588 DEGREE

```
CI vector
 =========

 222a0 22a 22a 22a      0.9287493
 22a20 2a2 22a 22a     -0.1434252
 22a20 22a 2a2 22a     -0.1434252
 222a0 2a2 2a2 22a     -0.1140635
 222a0 22a 2a2 2a2      0.0994775
 222a0 2a2 22a 2a2      0.0994775
 22a20 22a 22a 2a2      0.0942582
 22a20 2a2 2a2 22a      0.0619351
 a2220 22a 2a2 22a      0.0537280
 a2220 2a2 22a 22a     -0.0537280

 TOTAL ENERGIES                     -1917.26173609
```

```
NATURAL ORBITALS
 ================

  Orbital  Occupation    Energy     Coefficients
    6.1     2.00000    -0.71309     2 2px   0.66873     2 2py  -0.66873
    7.1     1.99023    -0.91995     1 3d2+  0.98523
    8.1     1.98920    -0.79314     2 2px   0.45053     2 2py   0.45053     2 2pz   0.51182
    9.1     1.93639    -0.76104     1 3d0   0.48061     2 2px  -0.33807     2 2py  -0.33807     2 2pz   0.62349
   10.1     1.08083    -0.51771     1 3d0  -0.84843     2 2pz   0.40974
   11.1     0.02222     0.46338     1 1s    0.92566     1 1s    0.49235     1 1s    0.26378     2 1s   -0.31180     2 2px   0.56360
                                    2 2py   0.56360     2 2pz   0.61008

    5.2     2.00000    -0.70338     1 2px   0.26418     2 2px  -0.51530     2 2py   0.78819
    6.2     1.99343    -0.69600     1 2px  -0.29102     2 2pz  -0.89055
    7.2     1.93546    -0.79138     1 2px   0.28153     1 3d1- -0.56177     2 2px   0.61155     2 2py   0.38604
    8.2     1.06590    -0.45795     1 2px   0.25099     1 3d1-  0.80276     2 2px   0.49415     2 2py   0.27129

    5.3     2.00000    -0.70338     1 2py   0.26418     2 2py  -0.51530     2 2px   0.78819
    6.3     1.99343    -0.69600     1 2py  -0.29102     2 2pz  -0.89055
    7.3     1.93546    -0.79138     1 2py   0.28153     1 3d1+ -0.56177     2 2py   0.61155     2 2px   0.38604
    8.3     1.06590    -0.45795     1 2py   0.25099     1 3d1+  0.80276     2 2py   0.49415     2 2px   0.27129

    5.4     2.00000    -0.68327     2 2py   0.68645     2 2px  -0.68645
    6.4     1.99282    -0.72103     1 2pz   0.49361     1 2pz  -0.40711     2 2pz  -0.36711     2 2py   0.57856     2 2px   0.57856
    7.4     1.95361    -0.76454     1 2pz  -0.28866     1 3d2-  0.46449     2 2pz  -0.75482
    8.4     1.04511    -0.43054     1 3d2-  0.86339     2 2pz   0.46483
```
